# Clarithromycin and endoscopic sinus surgery for adults with chronic rhinosinusitis with and without nasal polyps: study protocol for the MACRO randomised controlled trial

**DOI:** 10.1186/s13063-019-3314-7

**Published:** 2019-04-29

**Authors:** Carl Philpott, Steffi le Conte, David Beard, Jonathan Cook, William Sones, Steve Morris, Caroline S. Clarke, Mike Thomas, Paul Little, Jane Vennik, Valerie Lund, Helen Blackshaw, Anne Schilder, Stephen Durham, Spiros Denaxas, James Carpenter, James Boardman, Claire Hopkins, On behalf of the MACRO programme team, Neil Med, Keith Boland, Jane Woods, Teresa Ferreira

**Affiliations:** 1 Norwich Medical School, Chancellor’s Drive, University of East Anglia, Norwich, UK; 2 ENT Department, James Paget University Hospital NHS Foundation Trust, Great Yarmouth, UK; 3 SITU, University of Oxford, Oxford, UK; 4 Research Department of Primary Care and Population Health, University College London, London, UK; 5 Department of Applied Health Research, University College London, London, UK; 6 University of Southampton, Southampton, UK; 7 UCL Ear Institute, University College London, London, UK; 8 evidENT, UCL Ear Institute, University College London, London, UK; 9 Faculty of Medicine, National Heart & Lung Institute, Imperial College London, London, UK; 10 Institute of Health Informatics, University College London, London, UK; 11 London School of Hygiene and Tropical Medicine, University College London, London, UK; 12 Fifth Sense, Sanderum House, 38 Oakley Road, Chinnor, Oxfordshire, OX39 4TW UK; 13 ENT Department, Guy’s and St Thomas’ NHS Foundation Trust, London, UK

**Keywords:** chronic rhinosinusitis, endoscopic sinus surgery, clarithromycin, randomised controlled trial

## Abstract

**Background:**

Chronic rhinosinusitis (CRS) is a common source of ill health; 11% of UK adults reported CRS symptoms in a worldwide population study. Guidelines are conflicting regarding whether antibiotics should be included in primary medical management, reflecting the lack of evidence in systematic reviews. Insufficient evidence to inform the role of surgery contributes to a fivefold variation in UK intervention rates. The objective of this trial is to establish the comparative effectiveness of endoscopic sinus surgery (ESS) or a prolonged course of antibiotics (clarithromycin) in adult patients with CRS in terms of symptomatic improvement and costs to the National Health Service compared with standard medical care (intranasal medication) at 6 months.

**Methods/design:**

A three-arm parallel-group trial will be conducted with patients who remain symptomatic after receiving appropriate medical therapy (either in primary or secondary care). They will be randomised to receive: (1) intranasal medication plus ESS, (2) intranasal medication plus clarithromycin (250 mg) or (3) intranasal medication plus a placebo. Intranasal medication (current standard medical care) is defined as a spray or drops of intranasal corticosteroids and saline irrigations. The primary outcome measure is the SNOT-22 questionnaire, which assesses disease-specific health-related quality of life. The study sample size is 600. Principal analyses will be according to the randomised groups irrespective of compliance. The trial will be conducted in at least 16 secondary or tertiary care centres with an internal pilot at six sites for 6 months.

**Discussion:**

The potential cardiovascular side effects of macrolide antibiotics have been recently highlighted. The effectiveness of antibiotics will be established through this trial, which may help to reduce unnecessary usage and potential morbidity. If ESS is shown to be clinically effective and cost-effective, the trial may encourage earlier intervention. In contrast, if it is shown to be ineffective, then there should be a significant reduction in surgery rates. The trial results will feed into the other components of the MACRO research programme to establish best practice for the management of adults with CRS and design the ideal patient pathway across primary and secondary care.

**Trial registration:**

ISRCTN36962030. Registered on 17 October 2018.

**Electronic supplementary material:**

The online version of this article (10.1186/s13063-019-3314-7) contains supplementary material, which is available to authorized users.

## Background

### Background and rationale

#### The problem being addressed

Chronic rhinosinusitis (CRS) is a common source of ill health; 11% of UK adults reported CRS symptoms in a worldwide population study [1]. Symptoms, including nasal obstruction, nasal discharge, facial pain, anosmia and sleep disturbance, have a major impact on quality of life (QoL), reportedly greater in several domains of the SF-36 than angina or chronic respiratory disease [2]. Acute exacerbations, inadequate symptom control and respiratory disease exacerbation are common. Complications are rare but may include visual impairment and intracranial infection. Longitudinal primary-care electronic health records from the Clinical Practice Research Datalink, a nationally representative subset of primary-care data across consenting general practices, show that 1% of UK adults receive treatment for CRS from their general practitioner (GP) each year, with a median of four GP visits. They receive multiple medications, with 91% receiving an antibiotic prescription [3]. Secondary-care electronic health records for 2012/13 from Hospital Episode Statistics, England’s national administrative billing dataset for hospitals, show that approximately 40,000 sinus operations were performed in England and Wales, in addition to an estimated 120,000 outpatient consultations [4]. A worldwide study demonstrated that one in three CRS patients in primary care have poorly controlled symptoms [5]. The socio-economic cost of CRS is significant, with 57% of patients reporting absenteeism and 28% experiencing associated anxiety and depression [6, 7]. US businesses report CRS as one of the top 10 illnesses that cause absence from work [8].

#### Need and rationale for research in this area

The European Position Paper on Rhinosinusitis and Nasal Polyps (EPOS 2012) includes treatment guidelines and a research strategy for CRS, emphasising where limited evidence restricts care [9]. Key elements of this strategy are addressed herein, including the paucity of randomised controlled trials (RCTs) of treatments for rhinosinusitis. In the UK, there are no specific national treatment guidelines and adherence to EPOS 2012 is variable [10]. A recent ENT-UK commissioning guideline [11] acknowledges the lack of high-quality trials and although it does not recommend routine antibiotic use for CRS in primary care, GPs often prescribe repeated courses [12], which may cause resistance. There is growing interest in the immune-modulating and anti-inflammatory effects of macrolide antibiotics in treating chronic airway inflammatory disease, with low-dose long-term macrolides being prescribed for their immune response and not primarily as anti-bacterial agents [13]. Longer-term antibiotic use in secondary care has a low-grade recommendation, reflecting conflicting evidence from two RCTs [14, 15], resulting in a call for further trials [16–18].

First-line therapy is often delivered in primary care, and consists of initial treatment with intranasal corticosteroids, short courses of antibiotics or saline rinses. At least one in three CRS patients attending ear, nose and throat (ENT) clinics are considered to have failed this initial treatment, which is known as appropriate medical therapy (AMT), and are considered for endoscopic sinus surgery (ESS) [19, 20]. Insufficient evidence to define the role of surgery contributes to a fivefold variation in intervention rates across England, as determined by the clinical commissioning group [11]. The duration of symptoms before surgery varies from under 1 to over 10 years [21, 22]. If surgery is less effective than AMT, patients may be exposed to unnecessary risks and morbidity. If surgery is more effective, current delays reflect suboptimal patient care. Such uncertainty resulted in the inclusion of ESS in the Database of Treatment Uncertainties (DUETS) of the National Institute for Health and Care Excellence (NICE) [23].

As the EPOS 2012 guidelines recommend AMT prior to surgery, any trial must ensure patients have failed to respond to an adequate attempt at treatment using AMT. Intranasal corticosteroids and saline irrigation, for which there are strong recommendations for use based on high-quality level 1 evidence, should be included in AMT. Guidelines are conflicting regarding whether antibiotics should be included in primary medical management, reflecting the lack of evidence noted above. Our trial uses a pragmatic approach in which eligibility for surgery is based on a shared decision between the principal investigator (PI) and the patient, after initial treatment with AMT, in keeping with current guidelines [9, 24, 25].

#### Past and current research

Cochrane reviews have recently been completed covering the treatment options being assessed in this trial [26–31]. They support the use of intranasal corticosteroids and saline irrigations as standard treatment but express the need for more trials with antibiotics. A systematic review of ESS commissioned by the Human Tissue Authority identified the need for high-quality studies comparing surgery with medical treatment [32, 33]. Updated reviews of the literature and trial registries have not identified new studies. One potentially relevant trial reported by DUETS was never started. Two 2014 Cochrane systematic reviews of medical and surgical management concluded that further studies are urgently needed [34, 35]. There is little ongoing research that overlaps with the MACRO programme. Two relevant antibiotic trials are currently recruiting. A trial aiming to determine the optimum duration of longer-term antibiotics in patients with CRS [36] compared the effectiveness of 3 with 6 weeks of azithromycin but did not include a placebo arm. A commercially sponsored study compared azithromycin with a placebo in patients with persistent symptoms after ESS [37], but for many patients the aim was to avoid primary surgery [38]. Therefore, a trial to determine the effectiveness of antibiotics compared with ESS is essential. Finally, an RCT comparing ESS with topical medical therapy versus topical medical therapy alone for a subgroup with CRS with nasal polyps (CRSwNPs) is underway in the Netherlands [39].

#### Importance of the research

Despite the burden of CRS, UK health-economic evaluations are lacking. An American model demonstrates a high economic burden to patients, health-care systems and society [40, 41]. In 2011, CRS cost the US health-care system $8.6 billion with significant direct and indirect costs. Antibiotic resistance is considered one of the most significant threats to patient safety in Europe [42]. Evaluating the effectiveness of antibiotics and promoting appropriate usage is integral to the UK 5-year antimicrobial resistance strategy [43]. Given the high prevalence of CRS and the variability in prescribing antibiotics, this may represent a public health danger through selective pressure on bacteria and antibiotic resistance. In addition, the potential cardiovascular side effects of macrolide antibiotics have recently been highlighted [44]. The effectiveness of antibiotics must be urgently established to reduce unnecessary use and potential morbidity. International guidelines suggest that patients should receive the ‘maximum medical therapy’, which usually includes nasal (and possibly oral) steroids, antibiotic treatment and regular nasal douching, prior to surgical intervention. However, the use and duration of antibiotic use is very variable. In total, 91% of CRS patients currently receive an antibiotic prescription prior to referral. Recently, some clinical commissioning groups have insisted on a 3-month trial of macrolide antibiotics prior to secondary-care referral [48]; however, evidence to support this recommendation is lacking.

There is both wide variation in surgical practice and very high rates of revision surgery [45, 46]. There is a risk that patients may undergo surgery and its attendant complications without due reason. In contrast, recent changes to commissioning that restrict access to sinus surgery may deny beneficial treatment to patients. The risk is that limited evidence for effectiveness is assumed to indicate non-effectiveness, leading to the decommissioning of an effective treatment by clinical commissioning groups, with some already deviating widely from NICE commissioning guidelines [47]. It is essential to assess rigorously the effectiveness and cost-effectiveness of surgery to enable robust decision-making from patient, health service and societal perspectives. In non-randomised studies, surgery achieves significant improvements in health-related QoL and reductions in ongoing health-care utilisation. Pre- and post-trial health economic modelling will ensure the trial design reflects current pathways. If ESS is clinically effective and cost-effective, the trial may encourage earlier intervention. In contrast, if it is shown to be ineffective, there should be significant reductions in surgery rates.

The MACRO trial forms part of a larger programme of work, the overarching aims of which are (1) to address the major deficiencies in the evidence base for CRS management, (2) to establish best practice for the management of adults with CRS and (3) to design the ideal patient pathway across primary and secondary care.

### Objectives

#### Primary objective

The primary objective is to establish the comparative effectiveness of a prolonged course of antibiotics (clarithromycin) or ESS in adult patients with CRS in terms of symptomatic improvements and costs to the National Health Service (NHS), compared with standard medical (intranasal medication) care at 6 months.

#### Secondary objectives

The secondary objectives are:

- To measure clinical effectiveness using subjective self-report ratings and objective clinical measures
- To compare the clinical effectiveness according to phenotype: CRS with and without polyps (CRSwNPs versus CRSsNPs)
- To record the incidence and details of adverse events (AEs) in all treatment arms that are related to the trial medication or surgery intervention
- To establish the cost-effectiveness and cost utility of each arm relative to the others over the 6-month trial
- To embed a mixed-methods evaluation into the main trial to identify factors and processes necessary in the implementation of the trial findings

#### Objectives of internal recruitment pilot phase

The objectives of the internal recruitment pilot phase are:

- To randomise 72 patients at six pilot sites within 6 months. Recruitment will be deemed successful if ≥75% of the number of patients expected are recruited (i.e. 54 patients).
- To undertake an embedded qualitative study (the MACRO Conversation Study) that will identify recruitment challenges and suggest changes to optimise recruitment during the main trial phase

### Trial design

#### Overall design

The MACRO trial is a multi-centre three-arm placebo controlled parallel-group RCT that aims to randomise 600 adult patients with CRS. Patients who remain symptomatic after receiving AMT as deemed suitable by the local PI or co-investigator (Co-I) (either in primary or secondary care) and who are considered suitable candidates for further treatment (including surgery) will be randomised on a 1:1:1 basis to receive:

1. Intranasal medication plus ESS within 6 weeks of randomisation (ESS group)
2. Intranasal medication plus an initial 2-week course of capsules with 250 mg of clarithromycin twice daily followed by a 10-week course of capsules with 250 mg of clarithromycin once daily (antibiotic group)
3. Intranasal medication plus an initial 2-week course of placebo capsules twice daily followed by a 10-week course of placebo capsules once daily (placebo group)

Intranasal medication (current standard medical care) is defined as a spray or drops of intranasal corticosteroids and saline irrigations as per local formulary guidelines. Saline rinses will be provided by a research nurse (RN). These are non-investigational medicinal products (NIMPs) (see Name and description of each NIMP).

#### Internal recruitment pilot phase

For the 6-month internal recruitment pilot phase to begin, at least three sites should be open. Six sites in total will be included. An embedded qualitative study (the MACRO Conversation Study) will be conducted as part of the pilot phase to identify any challenges to recruitment. The qualitative work will involve audio-recording of recruitment consultations, and in-depth interviews with trial staff and patients who have been approached about participating in the MACRO trial. The outcomes of the qualitative work will be used to inform any changes to the design or conduct of the study, and to identify recruitment strategies or training needs to optimise recruitment for the main trial phase. If recruitment is lower than expected, we will propose a plan to increase recruitment based on the findings from the MACRO Conversation Study. The progress of this phase will be communicated regularly with the funder.

#### Main trial phase

If the pilot phase is successful, further pre-identified sites will be opened, and all sites will recruit the remaining participants required to achieve a sample size of 600 within the funded timescale of the MACRO trial (estimated total trial duration is 52 months). In terms of follow-up, work performed by both the research team [46, 48] and independent researchers [49] has shown that improvements in QoL remain stable between 6 and 60 months. Soler concluded that for clinical trials incorporating QoL outcomes, 6 months is considered to be an appropriate primary end point [49] and therefore, 6 months is the primary end point for this study. Subject to additional funding, it is proposed to continue the trial follow-up for a further 40–60 months after the initial 52 months’ trial duration, to enable the completion of long-term follow-up for all participants who have given their consent (up to 5 years).

## Methods/design

### Study setting

The trial will be conducted in at least 16 secondary or tertiary care centres around the UK. The PI at each site will be a dedicated consultant rhinologist. Details of sites can be found on the trial website: https://workstream2.themacroprogramme.org.uk/

### Participants

#### Eligibility of trial participants

Adult patients with CRS for whom symptom control has not been achieved following previous AMT and who are considered suitable candidates for further treatment (including surgery) will be assessed for eligibility, which must be assessed by a medically qualified doctor. Only personnel formally delegated by the PI to assess eligibility in the trial-specific delegation log may perform this task.

#### Trial participant inclusion criteria

Participants can be included if they meet all the following criteria:

- Adults aged 18 and over with a diagnosis of CRS according to European guidelines:
  - A minimum of 12 weeks’ history of inflammation of the nose and paranasal sinuses characterised by two or more symptoms, one of which should be either (1) nasal blockage, obstruction or congestion or (2) nasal discharge (anterior or posterior nasal drip). Other symptoms may include facial pain or pressure and reduction or loss of smell.
- Nasal endoscopy within the last 3 months to determine CRS diagnosis and phenotype (CRSwNPs or CRSsNPs)
- Non-contrast computed tomography (CT) scan within the last 12 months to determine the Lund–Mackay score and confirm suitability for ESS
- Moderate or severe symptoms within the last 3 months: SNOT-22 score ≥ 20
- Symptom control not achieved following previous AMT, as deemed by the local PI or Co-I, and therefore considered eligible for ESS
- Knowledge of English sufficient to understand written and verbal information about the trial, its consent process and the study questionnaires

#### Trial participant exclusion criteria

Participants will be excluded if they meet any of the following criteria:

- Lund–Mackay non-contrast CT scan score < 4
- Macrolide antibiotic treatment for >3 continuous weeks within the last 12 months
- ESS in previous 6 months or visible, open sinus cavities from previous surgery
- Taking maintenance oral steroids or biologics within the last 3 months
- Rare or complex sinus conditions, such as CRS secondary to systemic disease (e.g. ciliary dyskinesias or granulomatous diseases) or suspected malignancy
- Allergic fungal rhinosinusitis confirmed or suspected on CT imaging (expansion and mixed density opacification) necessitating immediate surgery
- Severe asthma (taking high doses of inhaled steroids, i.e. >1.5 mg per day)
- Females who are pregnant or breastfeeding
- Females of reproductive potential not prepared to use a reliable means of contraception (e.g. a hormonal contraceptive patch, intrauterine device, physical barrier or abstinence, if that is the preferred and usual lifestyle of the patient) at trial entry
- Females wanting to start a family during the initial 3 months of the trial
- Known immunodeficiency states including HIV and selective and multiple antibody deficiency states
- Severe septal deviation preventing endoscopic examination
- Contraindications to surgery (such as a significant medical co-morbidity)
- Any absolute contraindications to clarithromycin: risk factors to be assessed at screening include history of ischaemic heart disease, prolonged QT interval on an electrocardiogram (ECG), diabetes and aged over 65, or any medications known to interact with clarithromycin unless these can be discontinued during the 3 months of clarithromycin or placebo treatment
- Known allergies to the investigational medicinal product (IMP) and excipients of the IMP or the placebo
- Inability to give consent (significant cognitive impairment or language issues) or to understand and comply with trial instructions
- Participation in another trial in the past 4 months

#### Pre-trial routine assessments to assess eligibility

**Fig. 1:**
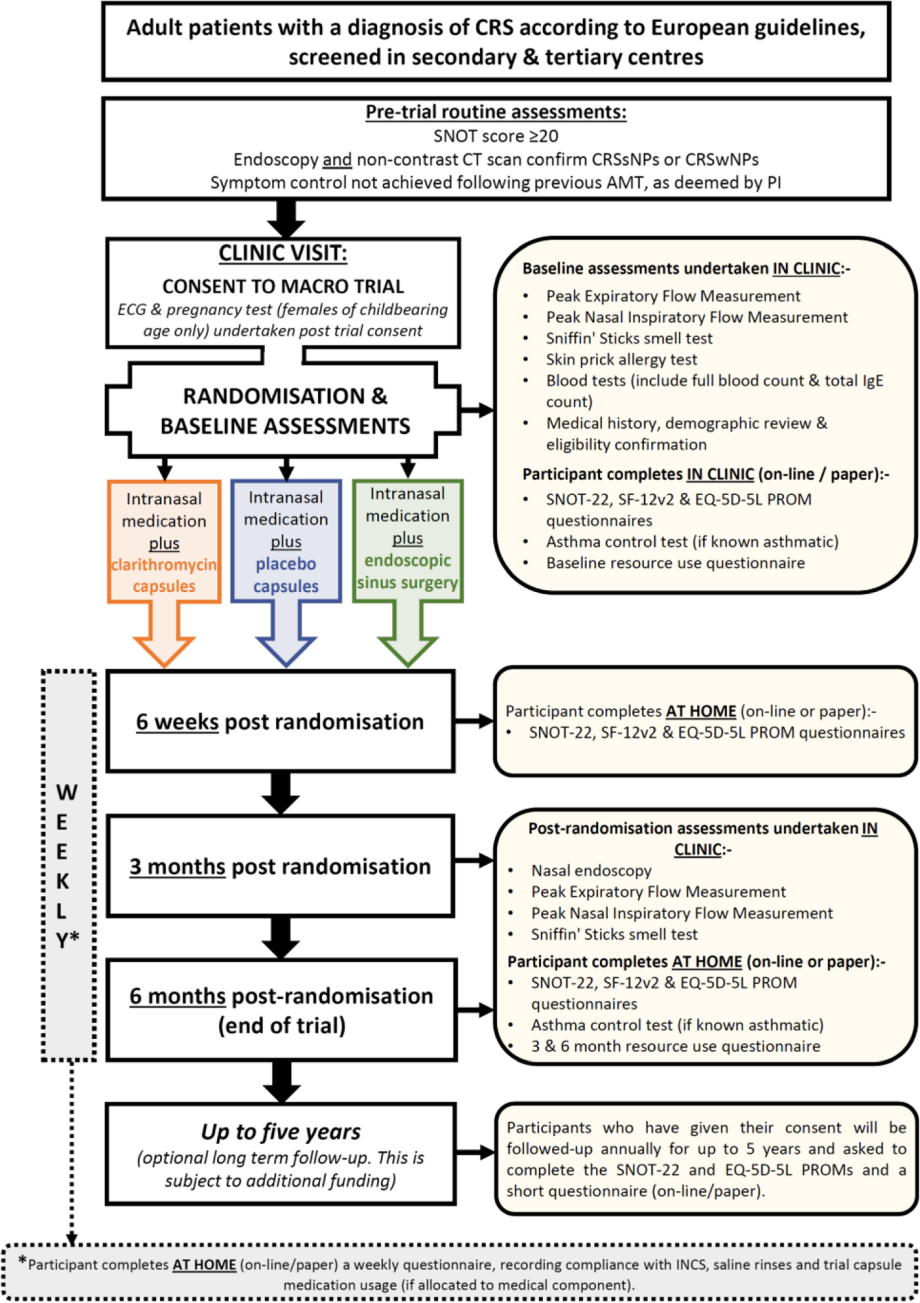
In our proposed setup, an automatic body representation is created based on which calculations for movement parameters can be automatically performed, for example step length, step width, joint angles and walking speed

All patients seen in clinic by the research team will undergo a routine nasal endoscopy to determine their CRS diagnosis and phenotype (CRSwNPs or CRSsNPs). The patients will also be asked to complete a routine SNOT-22 questionnaire, which is a validated patient-reported outcome measure (PROM), to determine the severity of symptoms (score ≥20 needed for eligibility). Any previous medical therapy will be discussed, and if symptoms have persisted following AMT (as deemed by the PI or Co-I) and the patient is considered suitable for further treatment (including surgery), a routine CT scan will be requested to confirm CRS and determine the Lund–Mackay score. The screening, consent and randomisation flow diagram is shown in Fig. 1.

### Interventions

#### Name and description of IMPs

UK-licensed 250 mg tablets of standard release clarithromycin will be over-encapsulated and provided in two bottles with a blinded label compliant with annex 13 of the EU GMP guidelines. The first bottle will contain the induction dose (one capsule to be taken orally twice daily for 2 weeks) and the second bottle will contain the remaining capsules, to be taken orally once a day for a further 10 weeks. The placebo capsules will also be provided in matching bottles with randomised, blinded labels.

#### Choice of antibiotic for the trial

With respect to the choice of antibiotic, we selected a macrolide, specifically clarithromycin, for several reasons:

- It has both anti-inflammatory and antimicrobial properties as demonstrated in other respiratory tract disorders [50–52]. At a dose of 250 mg orally twice daily, the peak tissue levels 4 h after administration have been shown to be 8.32 mg/kg ± 2.57 in nasal mucosa, with the drug characteristic being therapeutic serum concentrations and high tissue concentrations [53]. As a result, clarithromycin exhibits its immunomodulatory effects through inhibition of neutrophilic inflammation and macrophage activation [54].
- It has good action for typical CRS flora [55]. A dose of 250 mg twice daily is effective in the treatment of respiratory tract organisms, including *Moraxella catarrhalis*, *Streptococcus pneumoniae*, *Staphylococcus aureus* and *Haemophilus influenzae* (the minimum inhibitory concentration for the 90th percentile of the isolates is 0.064 mg/L) [56].
- It is currently recommended (Grade C recommendation) by EPOS and the ENT-UK rhinosinusitis commissioning guidelines for selected patients with CRS [9, 57]
- Although EPOS states that its use should be restricted to patients who have confirmed CRS on endoscopic examination, it is increasingly being recommended that GPs prescribe a prolonged course to patients without confirmatory endoscopy in primary care; however, in the MACRO Programme Workstream 1, health informatics data showed little evidence that courses lasting longer than 3 weeks were being prescribed in primary care.
- Two RCTs have produced conflicting results for the efficacy of macrolides [14, 15], necessitating a trial that can differentiate according to phenotype.
- Previous studies suggest a longer course of medication is better [58, 59], although in practice both duration and compliance vary. We propose to adopt a 12-week course of clarithromycin within this trial, giving this study a realistic timeline and recruitment targets.
- Systematic reviews have highlighted that the overall quality of evidence from previous studies is low due to limitations in trial designs. A firm conclusion regarding the effectiveness of macrolides for CRS could not be reached [26, 60].
- Several publications have raised concerns about the cardiac toxicity of erythromycin in patients with a prolonged QT interval [61, 62]. We have selected clarithromycin since: (1) erythromycin has poor tolerability, (2) a previous RCT showed that azithromycin has poor efficacy and (3) there is limited availability of roxithromycin in the UK.
- Aside from the issues above, clarithromycin is readily available and has a reasonable side-effect profile (as seen in our feasibility study) [63]

#### Name and description of each NIMP

A spray or drops of intranasal corticosteroids will be prescribed for all participants to use throughout the trial, as per local formulary practice. Saline irrigation packs will be provided by NeilMed® Pharmaceuticals. Both concomitant medications are licensed in the UK and used within their indication. These drugs are considered to be NIMPs in this trial. Host sites are responsible for maintaining a system that allows adequate reconstruction of NIMP movements; these will be recorded on the case report forms (CRFs)*.*

#### Concomitant medication

If there is an acute exacerbation of the condition, the participant may receive appropriate additional medical treatment as decided by the ENT surgeon or their GP. This can include oral steroids or full-dose broad-spectrum or culture-directed antibiotics. Details of these concomitant medications will be captured in the patient-reported resource-use diaries (to be completed at baseline and at 3 and 6 months). A list of medicinal products contraindicated in the use of IMP is available on request from the trial manager at macrotrial@nds.ox.ac.uk.

#### Endoscopic sinus surgery

ESS will be performed by consultant rhinologists according to the techniques described by Stammberger, Lund and Kennedy, with surgery proceeding in a stepwise fashion through polypectomy (where present), uncinectomy, middle meatal antrostomy, ethmoidectomy, with additional frontal and sphenoid surgery in selected participants. The extent of surgery to be performed will be decided at an individual participant level but recorded by the operating surgeon as part of the CRF (having first been documented in the medical notes). Instrumentation will not be standardised between centres. Surgery can be undertaken by a registrar under consultant supervision. As this is a pragmatic trial, there will be no specific further standardisation of the ESS procedure at each site.

#### Treatment schedule

See also the overall trial design described above. Participants will receive a prescription to take to the pharmacy for the spray or drops of intranasal corticosteroids and will receive a 3-month supply to last them until their next scheduled visit. Participants will also be supplied with sinus irrigation bottles and sachets provided by NeilMed Pharmaceuticals Inc (PO Box 2853, Coulsdon, Surrey, CR5 2WN, United Kingdom). Each site will have a designated pharmacy lead for the trial, who will be informed by the Surgical Interventions Trials Unit (SITU) at the University of Oxford of the allocation of each participant and their requirement for clarithromycin or a placebo.

If a participant does not receive surgery within 6 weeks of randomisation, this is not considered a protocol non-compliance. The date of surgery will be captured in the patients’ medical notes and then transcribed onto the treatment CRF from the medical records.

#### Dose modifications

There are no dose modifications in the MACRO trial. Should a patient be unable to tolerate the dose of medication, they will discontinue treatment but will asked to continue their participation in the trial (as discussed in the section on ‘Stopping trial treatment’). The dosage of clarithromycin is in line with an open-label study in the field [64]; 250 mg is a readily available dose. The initial regimen is for 2 weeks as this is considered to be therapeutic in terms of an antibiotic effect. Participants will then be requested to take 250 mg of clarithromycin once a day for a further 10 weeks as the key purpose of this medication in the MACRO trial is immunomodulatory [52]. A lower dose is suitable for this effect to be exerted and takes into consideration recent concerns around cardiovascular morbidity that were addressed by Workstream 1 as part of our trial consensus process.

#### Assessment of IMP and NIMP compliance

Compliance with medical treatment will be recorded in the weekly compliance diaries completed by the participants.

#### Stopping trial treatment

Participants allocated either to the active IMP or placebo will be requested to carry a card with them at all times, outlining that they are participating in the MACRO trial.

If a suspected serious interaction (or serious adverse event (SAE) that is deemed related to the study medication) occurs in a participant randomised to either clarithromycin or the placebo, the attending clinician, PI or Co-I should tell the participant to stop taking the trial medication immediately and inform the MACRO trial office using the secure SITU email address (situ.oxford@nhs.net) from an NHS email account.

If a participant stops taking their trial medication due to unwanted side effects, the participant must inform the PI, Co-I, RN or research practitioner (RP) as soon as possible. The PI, Co-I, RN or RP should then contact the trial office using the secure email address situ.oxford@nhs.net.

In both cases, the PI, Co-I, RN or RP should first document this in the medical notes, and then complete a change of status CRF detailing that the participant has stopped taking their trial medication and the reason for stopping. The CRF should be sent to the MACRO trial office. The participant will be requested to remain in the trial and to continue to undertake all follow-up activities, as per this protocol (even if they are unblinded to their study treatment). The patient should be asked to return any unused trial medication at their next follow-up visit.

If the participant is allocated to ESS and the treatment does not go ahead or is changed part way through (for clinical reasons), this should first be documented in the medical notes and a change of status CRF should be completed. The CRF should be sent to the MACRO trial office. The participant will be asked to remain in the trial and to continue to undertake all follow-up activities, as per this protocol.

A change of status CRF should be completed by the PI, Co-I, RN or RP and sent to the MACRO trial office if the patient’s treatment deviates from the allocated intervention. The participant will be requested to remain in the trial and to continue to undertake all follow-up activities, as per this protocol.

### Outcomes

The primary outcome is disease-specific health-related QoL using the SNOT-22 at 6 months.

The secondary outcomes are:

- Endoscopic score (Lund–Kennedy score)
- Grade of polyps (0–3, Lildholdt score)
- Health-related QoL and quality-adjusted life-years (QALYs), measured by the SF-12v2 and EQ-5D-5 L questionnaires
- Need for additional treatment (e.g. oral steroids, antibiotics, etc.)
- Olfactory function measured using Sniffin’ Sticks
- Upper and lower respiratory function, measured using peak expiratory flow rate and peak nasal inspiratory flow rate
- Asthma control test (participants with asthma only)
- AEs due to treatment
- Health-care resource use, including medications and visits to primary and secondary care, recorded using patient questionnaires
- Number of days of work missed, recorded using patient questionnaires
- Overall cost and incremental cost per QALY gained, from the cost perspective of the NHS and personal social services, calculated using QoL scores
- Budget impact of treatment

### Participant timeline

See details of the treatment schedule above and the participant flow chart in Fig. 1. All participants will be enrolled in the trial for 6 months from randomisation. Additional file 1: Appendix 2 shows the visit schedules.

### Visit schedule: baseline and follow-up assessments

Once informed consent has been obtained and the ECG and pregnancy test (where applicable) have confirmed the participant’s eligibility, a comprehensive baseline assessment will be undertaken:

- Clinical assessments:
  - o Peak expiratory flow measurement
  - o Peak nasal inspiratory flow measurement
  - o Sniffin’ Sticks olfactory test
  - o Skin prick allergy test (or RAST inhalant screen). Allergens to be tested for must include house dust mites, mixed grass, mixed tree, mixed mould, dog and cat. Specific allergens are acceptable in place of mixed tree, mixed grass or mixed mould.
- Blood tests, including full blood count and total immunoglobin E count (results from blood tests taken within last the 6 months are acceptable; however, the participant must not have taken prednisolone or other oral steroids within 6 weeks preceding the blood test)
- Eligibility confirmation
- Participant demographics and medical history

The results of these assessments will be recorded in the baseline CRF (and medical notes if clinically important) along with data from the routine care pre-screening assessments:

- Lund–Mackay score from the CT scan
- Lund–Kennedy score from the endoscopy
- Lildholdt polyp grade from the endoscopy
- SNOT-22 score

Participants will be requested to complete three baseline disease-specific and generic PROMs online during the baseline clinic visit (the SNOT-22, SF-12v2 and EQ-5D-5 L questionnaires), as well as an asthma control test, if they are known to be asthmatic. Participants will also be requested to complete an initial online baseline resource-use questionnaire, which will ask them about their use of health-care services over the last 3 months before joining the trial. These questionnaires will be completed online by the participant during the baseline visit so that a RN or RP is on hand to answer any questions. If any site has difficulties with offering these questionnaires online, a paper version is also available.

Following the initial baseline assessment, all participants in all treatment arms will be followed up in clinic at 3 and 6 months post-randomisation (±7 days). The following measurements will be undertaken and recorded on a trial-specific follow-up CRF:

- Nasal endoscopy
- Peak expiratory flow measurement
- Peak nasal inspiratory flow measurement
- Sniffin’ Sticks olfactory test

Women of childbearing potential will not be requested to undertake another pregnancy test at their 3- or 6-month post-randomisation clinic visit. However, the site should reiterate to the woman that if pregnancy occurs, a member of the local site staff should be informed immediately, and a pregnancy reporting form should be completed and sent to the central MACRO office.

Participants will be contacted separately by the MACRO trial office and requested to complete the SNOT-22, EQ-5D-5 L and SF-12v2 PROM questionnaires electronically at 6 weeks, 3 months and 6 months post-randomisation. Participants will also be asked to complete an asthma control test (if they are known to be asthmatic) at 3 and 6 months. Those participants who state at recruitment that they cannot or do not wish to complete these questionnaires online will be sent the paper version by the MACRO trial office and requested to complete the questionnaires and to return them in a prepaid envelope.

Participants will also be asked to complete a weekly questionnaire (recording their compliance with intranasal and placebo or clarithromycin trial-specific medication usage) and a separate health economics resource-use questionnaire at baseline, and at 3 and 6 months (recording other medication and health-care resource use, and details of time off work and other costs) to evaluate:

- Need for additional treatment (e.g. oral steroids, antibiotics, etc.)
- Adverse effects of treatment
- Health-care visits to primary and secondary care
- Number of fays of work missed
- Cost and cost-effectiveness from the perspective of the NHS and personal social services
- Compliance with the trial medication

A schedule of all trial assessments and procedures is set out in Additional file 1: Appendix 2. Samples will be taken for a full blood count and total immunoglobulin E and will be carried out at local laboratories.

### Sample size

The trial will recruit 600 participants. Recruiting participants from 17 centres (therefore, with 17 ESS surgeons) is considered a realistic assumption for the following sample size calculations. The sample size is justified based upon achieving at least 80% statistical power at the two-sided 5% significance level. No adjustment for multiple comparisons has been made, as each of the treatment comparisons are distinct. The minimum clinically important difference has been estimated to be about 8.9 points based upon an anchor study [65]. A 10-point difference in SNOT-22 (0.5 standard deviations (SD), Cohen’s effect size assuming an SD of 20) is often considered a medium-sized effect size and an important difference for this type of outcome. A previous study suggests that a larger effect for surgery against an alternative treatment is plausible, as large as 13.8 [31]. Using target differences of and 8.9 and 10 (and SD of 20) would require and 107 and 90 per group (331 and 270 overall) to achieve 90% statistical power at the two-sided 5% significance level. Offsetting this is the possibility of clustering within the surgical arm, which affects pairwise comparisons involving surgery, and the need to perform a subgroup analysis for CRSwNPs versus CRSsNPs participants. Allowing for clustering (intraclass correlation coefficient of 0.05 and 17 clusters of equal size) in the surgical group would lead to 102 per group (306 overall) for a target difference of 10 points for 90% power. For this trial size and using a target difference of 8.9 but an otherwise identical calculation, there would be 80% power. To enable secondary analyses of treatment interactions by subgroups with or without polyps, the overall sample size was inflated to 600 (after allowing for 10% missing data). This size of study would allow us to detect a difference of 8.9 and 10 (SD of 20) to be detected in all CRS patients with >90% power after adjusting for clustering for the main comparisons involving the ESS group; this is the case even when allowing for the impact of potential variable cluster sizes (cluster size variance of 49). A study of 600 would also likely provide around 50% and 80% power for testing the treatment interaction by subgroup (CRSwNPs versus CRSsNPs) for a target difference of 10 and 13.8, respectively, after allowing for clustering in the surgical arm and an equivalent variation in cluster sizes.

### Recruitment

#### Participants in the internal pilot phase: MACRO Conversation Study

During the internal recruitment pilot phase, potential trial participants will be invited to take part in an embedded qualitative study (MACRO Conversation Study), which is designed to evaluate and optimise trial recruitment. All potential participants will receive a separate participant study information sheet (PIS) for the MACRO Conversation Study whilst waiting in the outpatient department (prior to being seen in the clinic).

#### All participants

Posters and flyers will be placed in outpatient departments with information about the MACRO trial. All potential trial participants will be seen in an outpatient clinic by the PI or Co-I and by the RN or RP, where the aforementioned routine screening assessments will be carried out and the MACRO trial will be verbally introduced by the PI or Co-I and RN or RP. The participant will be informed that in addition to the routine screening assessments, a trial-specific ECG scan (to exclude contraindications to clarithromycin) will be undertaken as well as a pregnancy test (urine test) if the potential participant is of childbearing potential. This information will be included in the main trial consent form.

If interested in the trial, the patient will be provided with a copy of the MACRO PIS to take home and review. The PIS will detail the exact nature of the study, what it will involve for the patient, the implications and constraints of the protocol, the known side effects and any risks involved in taking part. It will be clearly stated that the participant is free to withdraw from the study at any time for any reason without prejudice to future care, and with no obligation to give a reason for their withdrawal. In this event, the choice of treatment will be decided by the patient and their clinical team.

The RN or RP should update the screening log and place a MACRO sticker on the notes, to help identify the patient when they return to the clinic. All patients will be given sufficient time (a minimum of 48 h) to consider the trial and to decide if they would like to take part. Screening logs must be kept up-to-date and completed fully at all times. They are sent to the MACRO trial office for a monthly review.

If the nasal endoscopy and SNOT-22 questionnaire confirm that the patient is a potential MACRO participant but the patient has not received AMT as deemed by the PI or Co-I, the MACRO trial can still be introduced and the participant will be given a copy of the PIS to take home. If, following AMT, symptom control has not been achieved and further treatment is deemed necessary, the patient will be seen in the clinic once more and the MACRO trial discussed in depth.

### Assignment of interventions

#### Sequence generation

Following consent, once trial eligibility has been confirmed, participants will be randomised into the trial by the PI, Co-I, RN or RP. Participants will be randomly allocated to a treatment option using an automated web-based secure randomisation system (RRAMP) provided by the Oxford Clinical Trials Research Unit (OCTRU) with a 1:1:1 allocation ratio. The algorithm will stratify by the presence of polyps and centre using permuted blocks of varying size. The sequence will be generated by the trial statistician.

#### Allocation concealment mechanism and implementation

The centrally managed randomisation will ensure allocation concealment and prevent selection bias. The participant’s identifiable information will be recorded on the randomisation form and will be uploaded to a separate encrypted database at the University of Oxford. Participants allocated to receive the placebo or antibiotic will be assigned a treatment pack number for the corresponding medication. Treatment pack numbers will be randomly generated to ensure allocation concealment and blinding.

#### Blinding (masking)

Blinding of participants and medical staff will be maintained for the comparison of clarithromycin by using a placebo identical in appearance to the antibiotic, although the MACRO trial office can reveal the treatment allocation in case of clinical need. Participants and medical staff will not be blinded to receiving surgery. At the end of each trial participant’s follow-up period of 6 months, the participant will return to normal NHS care. Those who remain symptomatic at this point will receive further treatment as defined by their ENT clinician, which may include being offered steroids, antibiotics or ESS, depending on which arm of the trial they were in. Patients who were allocated to either the placebo or clarithromycin will not be told of their allocation at the end of their 6-month trial period, except in an emergency.

If there is a need to treat a patient in an emergency following a serious adverse reaction (SAR), the treating PI or medical doctor can break the code using the randomisation system to see if the patient was on the active drug. Under these exceptional circumstances, the PI or a member of the site team can unblind the participant using the clinical trial unit’s in-house RRAMP system. If unblinding is required for any other reasons apart from an emergency, site staff must submit a request (through RRAMP) and the request will be reviewed. If appropriate, it will be approved by a member of the central MACRO trial office. Where a SAR is not an emergency to treat but is deemed to be a suspected unexpected serious adverse reaction (SUSAR), then the clinical trial unit will break the code for reporting purposes without necessarily involving the PI. If on breaking the code it is found that the patient was actually taking the placebo, the event will not be classed as a SUSAR.

### Data collection methods

#### Confidentiality

All data will be handled in accordance with the General Data Protection Regulation (EU) 2016/679.

Participants joining MACRO will consent to giving identifiable information that will include their name, email address and telephone contact numbers to allow completion of PROMs and resource-use diaries electronically. If the participant does not consent to completing the documentation electronically, they will be asked instead to provide their home address and telephone number so that the MACRO trial office can send the PROM questionnaires and resource-use diaries by post. This is coordinated centrally by the MACRO trial office. Participants’ identifiable information will be kept securely in a University of Oxford network database, which is separate to the clinical database.

Upon randomisation, the data will be pseudonymised and a study number will be given to each participant. CRFs will not bear the participant’s name, and the study number will be used for identification. Screening logs will list the participant’s initials, year of birth and trial ID to allow identification by site research staff when they return to the clinic.

#### Data collection tools and source document identification

Clinical data will be collected from sites on trial-specific paper CRFs, which will be completed by the local research teams and sent to the MACRO office for data entry. The data will be entered into a validated installation of OpenClinica (www.openclinica.com). The data are held in a secure database and can only be accessed by authorised users via the OpenClinica application, which resides on a webserver hosted and managed by the IT Services Department of Oxford University’s Medical Services Division (http://www.imsu.ox.ac.uk/). Some CRF data may be entered electronically into LimeSurvey. Participants will be requested to complete PROM questionnaires and resource-use diaries electronically using LimeSurvey every week and every 3 months, respectively. A paper version will also be available for participants to complete, which can be returned using prepaid envelopes. Text messaging and email reminders to participants (who have consented to provide their telephone numbers and email addresses) who have not returned the completed PROM questionnaires and resource-use diaries will be used to maximise the completeness of the data. The MACRO trial office may also call participants if there are a number of outstanding PROM questionnaires and resource-use entries not completed by the participant.

If it becomes clear during the pilot phase that there is a low return rate for the prospective online weekly compliance diaries, the trial management group (TMG) will discuss whether to switch to collecting data retrospectively for this aspect of the trial, i.e. at the 6-week, 3-month and 6-month time points.

### Statistical methods

#### Statistical analysis

Full details of the statistical analysis plan will be agreed by the MACRO programme steering committee (PSC) before the data are unblinded. A summary of the planned analyses is presented here. The primary analysis will be according to the randomised allocation, irrespective of subsequent treatment compliance, and conducted at the two-sided 5% significance level with 95% confidence intervals. No adjustment for multiple comparisons is planned, as the comparisons relate to distinct clinical decisions (use or non-use of an antibiotic and surgery versus medical management of one form). Baseline data and participant flow information will be summarised without a formal analysis. The primary outcome will be analysed using linear regression adjusted for baseline SNOT-22 and also for the presence or not of polyps at baseline. Clustering by surgeon will also be accounted for where relevant using an appropriate method (e.g. the cluster robust option in Stata). Secondary outcomes will be analysed similarly using generalised linear models.

#### Sensitivity and other planned analyses

The impact of missing primary outcome data will be assessed in sensitivity analyses, e.g. the multiple imputation approach, as appropriate. Exploratory subgroup analyses will assess the impact of the presence of polyps under the treatment effect. The impact of compliance will be evaluated using a complier average causal effect approach or a similar approach.

#### Interim analysis

No interim analyses are anticipated prior to completion of the follow-up for the designated time points. This is due to the nature of the trial, the primary outcome and the desire for reasonable precision to assess the subgroups for CRSwNPs and CRSsNPs.

### Data monitoring

Details of the committee personnel can be found in Additional file 1: Appendix 1.

#### Trial management group

The TMG will include the chief investigator, lead collaborative investigator, trial staff and members of the MACRO programme. A member of the sponsor team will also be invited to attend. The TMG will be responsible for overseeing the trial. Monthly TMG teleconferences will take place at the start of the trial, and at least two face-to-face meetings will take place annually.

The TMG will review recruitment figures, SAEs and substantial amendments to the protocol prior to submission to the research ethics committee or the Medicines and Healthcare Products Regulatory Agency (MHRA). All PIs will be kept informed of substantial amendments through their nominated responsible individuals. There will be a TMG charter for the MACRO trial.

#### Trial steering committee

As MACRO forms part of a programme of work, there will be an overarching independent PSC for the duration of the programme. The PSC contains members experienced in each specialist area of the MACRO programme. Membership also includes an experienced triallist, a clinician, a statistician, a qualitative researcher, a health economist, a GP and a patient. During the MACRO trial, the PSC will assume the role of the trial steering committee (TSC) and will provide overall supervision of the trial. The TSC will review the recommendations of the independent data safety and monitoring committee (DSMC) and, on consideration of this information, recommend any appropriate amendments or actions for the trial as necessary. The TSC acts on behalf of the funders and sponsor.

#### Data safety monitoring committee

The role of the DSMC is to provide independent advice on data and the safety aspects of the trial. The committee will meet annually to review safety data and any other issues. There will be a DSMC charter for the MACRO trial.

#### Stopping rules

The trial may be stopped before completion for the following reasons:

- On the recommendation of the TSC or DSMC
- On the recommendation of the sponsor and chief investigator

#### Discontinuation and withdrawal of participants

##### Participant withdrawal from trial treatment

If a participant expresses their wish to withdraw from trial treatment, sites should explain the importance of their continuing to be followed up for the trial (including completing the patient-reported trial questionnaires and diaries) and seek permission to allow use of routine follow-up data for trial purposes. The importance of the safety follow-up should be emphasised to the participant in the PIS. If a participant decides to withdraw from treatment, this must be recorded in a change of status CRF and their medical notes. The participant may withhold their reason for withdrawal. However, if the participant gives a reason for their withdrawal from trial treatment, this should be recorded. If a participant allocated to either of the medical therapy arms requests to be given a non-trial treatment in addition to or instead of their trial treatment, this should also be recorded in the change of status CRF. If the participant wishes to withdraw from the trial follow-up, a discontinuation CRF should be completed. Where necessary for safety (e.g. if the participant requests to receive clarithromycin), they will be unblinded at this point. Permission to allow data collection to continue will be sought, irrespective of the treatment received.

##### Withdrawal of consent to data collection

If a participant explicitly states that they do not wish to contribute further data to the trial, their decision must be respected and recorded in the discontinuation CRF and their medical notes. Any data already collected on the patient will be included in analyses.

##### Loss to follow-up

If a participant moves from the area, every effort should be made to follow the participant up at another participating trial site and for this new site to take over the responsibility for the participant. If a participant is lost to follow-up at a site, every effort should be made to contact the participant’s GP to obtain information on their current status.

#### Replacements

Individuals who do not comply with the treatment allocation or the study protocol more generally (e.g. by not attending a clinical visit or completing a questionnaire) will not be replaced, as the trial is using an intention-to-treat analysis. Note that a randomisation error (e.g. incorrectly randomising an individual a second time) will not be considered a valid randomisation.

#### Definition of end of trial

The expected duration of recruitment and follow-up for the primary outcome data collection point is 52 months from recruitment of the first patient. For regulatory purposes, the trial will be deemed to have ended when all data have been received and cleaned and when all queries have been resolved at each site. Subject to additional funding, trial follow-up will continue for a further 40–60 months after the initial 52 months’ trial duration, to enable completion of the long-term follow-up for all participants who have given their consent (up to 5 years). For regulatory purposes, the trial will then be deemed to have ended when all the long-term follow-up data have been received and cleaned.

### Harms

The sponsor’s responsibility for pharmacovigilance has been delegated to SITU within OCTRU, University of Oxford. The severity of AEs (for either IMP or ESS) will be classified as mild (1), moderate (2) or severe (3), as described in Table 1.

**Table 1:** Categories for adverse events

Category	Definition
Mild	The adverse event does not interfere with the participant’s daily routine and does not require intervention; it causes slight discomfort
Moderate	The adverse event interferes with some aspects of the participant’s routine or requires intervention, but is not damaging to health; it causes moderate discomfort
Severe	The adverse event results in an alteration, discomfort or disability that is clearly damaging to health

If an event satisfies the definition of a SAE, the PI, Co-I, RN or RP should complete a paper SAE form. The following information will be recorded: description, date of onset and end date, severity, assessment of causality due to trial medication or ESS, other suspect drug or device, and action taken. Follow-up information should be recorded as necessary. Trial visits and hospitalisation as part of standard clinical care will not be reported as an SAE, unless the hospitalisation extends beyond the expected length. SAE forms must be sent via secure email to situ.oxford@nhs.net within 24 h of the local trial team becoming aware of the event.

Any SAE or any AE that is more severe than would be expected that is considered related to the trial medication or surgery intervention, as judged by a medically qualified investigator, will be followed, either until resolution or the event is considered stable.

#### Events related to the IMP

The most frequent and common adverse reactions related to clarithromycin therapy for adults are:

- Abdominal pain
- Diarrhoea
- Nausea
- Vomiting
- Dysgeusia
- Headache
- Insomnia
- Dyspepsia
- Rash
- Hyperhidrosis
- Abnormal liver function test

These adverse reactions are usually mild in intensity and are consistent with the known safety profile of macrolide antibiotics. AEs will be recorded in the hospital notes in the first instance. The clinical symptoms of all AEs will be recorded, accompanied by a simple and brief description of the event, including dates as appropriate.

Any AE or adverse reaction that occurs after the baseline assessment (whether as would be expected, more severe than would be expected or unexpected) should be recorded. If an event is deemed to be more severe than would be expected, the PI, Co-I, RN or RP must complete an AE CRF, otherwise it should be detailed in the follow-up CRF at the 3- or 6-month clinic visit when the patient is seen by the PI, Co-I, RN or RP.

#### Events related to ESS

The following AEs are possible following ESS:

- Post-operative bleeding needing nasal packing or readmission (1 in 200)
- Post-operative infection requiring antibiotics (1 in 15)
- Bruising around the eye (1 in 500)
- Leakage of cerebrospinal fluid (due to damage to the skull base, which may lead to meningitis) requiring repair, either at the time of surgery and delaying the patient’s discharge or later and causing a re-admission (1 in 1500)
- Major orbital injury leading to double vision or blindness (<1 in 10,000)

These events, and all other events relating to the ESS, will be recorded on the post-procedure CRF. Events occurring after the baseline assessment should be detailed in the 3- and 6-month post-randomisation follow-up visit CRF by the PI, Co-I, RN or RP.

Any leak of cerebrospinal fluid leading to meningitis (and hospitalisation) or major orbital injury leading to double vision or blindness must be recorded as an SAE. A delayed discharge for participants undergoing ESS will not be considered an SAE if the participant’s hospital stay is less than 24 h.

### Auditing

#### Processing of SAE forms and assessment of expectedness

The trial office will process SAEs as per the instructions in OCTRU’s relevant standard operating procedure for safety. SAEs will be reviewed by a nominated person and any queries clarified with the site. The nominated person will also perform a central assessment of expectedness. For SAEs related to the IMP or placebo, expectedness will be assessed according to the current approved summary of the product characteristics for clarithromycin.

##### SUSAR reporting

All SUSARs will be reported by SITU to the relevant competent authority (MHRA) and to the research ethics committee and other parties as applicable. For fatal and life-threatening SUSARs, this will be done no later than 7 calendar days after the sponsor or delegate is first made aware of the reaction. Any additional relevant information will be reported within 8 calendar days of the initial report. All other SUSARs will be reported within 15 calendar days. PIs will be informed of all SUSARs for the relevant IMP for all studies using the same IMP with the same sponsor, whether or not the event occurred in the current trial. Line listings will be sent to sites every 3 months and any new SUSARs will be discussed at each TMG meeting.

##### Development safety update reports

SITU, on behalf of the chief investigator and sponsor, will submit (in addition to the expedited reporting noted above) development safety update reports once a year throughout the clinical trial, or on request, to the competent authority (MHRA in the UK), the relevant ethics committee, the Health Research Authority (where required) and the sponsor. The report will be submitted within 60 days of the developmental international birth date of the trial each year until the trial has been declared as ended.

##### Notification of deaths

In the unlikely event of a death, the site must complete a notification of death form and send it to the trial office within 24 h of the site becoming aware of the event. If the death is the outcome of an SAE, then it must be recorded on both an SAE form (within 24 h) and on a notification of death form.

##### Pregnancy

Female participants of childbearing potential will be counselled appropriately by the PI or Co-I during the clinic visit and they will be asked to use a reliable method of contraception (e.g. hormonal contraceptive patch, intrauterine device, physical barrier or abstinence, if that is the preferred and usual lifestyle of the patient) for the duration of the trial.

##### IMP and placebo arms

If a female participant becomes pregnant during the 3-month active IMP or placebo period, a completed trial-specific pregnancy reporting form should be sent to the secure email address situ.oxford@nhs.net within 24 h of the site becoming aware of the event. The local PI will respond to any queries raised by the trial office as soon as possible. The participant should be unblinded and told to stop taking the medication, but will be asked to remain in the study, and they will be followed up as per the protocol. The site should complete a change of status CRF and send it to the MACRO trial office.

If a female participant becomes pregnant outside of the 3-month active or placebo period, the site must complete a pregnancy notification form and submit it to the trial office. No further action is required by the site.

##### ESS arm

If a female participant becomes pregnant before receiving ESS, the participant should be withdrawn from trial treatment, but will be asked to remain in the trial and to undergo trial follow-up as per the protocol. The site should complete a discontinuation CRF and a change of status CRF and send these to the MACRO trial office.

If a female participant becomes pregnant after receiving ESS, the site must complete a pregnancy notification form and submit it to the trial office. No further action is required by the site and the participant will be followed up for the trial as per this protocol.

##### Overdoses

Sites will record details of reported overdoses of the IMP or the placebo on the deviation log and inform the trial manager at OCTRU as soon as possible after being made aware of the information. An overdose is defined as three or more tablets of the IMP or placebo in 24 h for 1 day or longer. If an overdose has taken place, the patient will suspend medication for at least 48 h, and resume the previous dose as soon as possible afterwards, at the discretion of the PI. If an AE occurs and it is deemed to be more severe than expected (see recording of adverse events above), the site must complete an AE CRF. If an SAE has not occurred, the patient may continue in the trial.

If the event satisfies the definition of a SAR, the site must also complete an SAE form and the SAE reporting procedure should be followed. The trials office will notify the sponsor that an overdose associated with a SAE has occurred.

### Ethics and dissemination

#### Protocol amendments

The sponsor will ensure that the trial protocol including any agreed amendments, PIS, consent form, GP letter and supporting documents have been approved by the appropriate regulatory body (MHRA in UK) and an appropriate research ethics committee, prior to any participant recruitment. Amendments will not be implemented prior to receipt of the required approvals.

#### Consent

Informed consent will be obtained from all participants. Each potential participant will be telephoned by the RN or RP at least 48 h after being given the MACRO PIS. The RN or RP will answer any initial questions about the trial over the phone, and also invite the patient to return to the clinic to discuss their possible participation in the MACRO trial in more depth. The medical notes must record when and what version of the PIS was given to the participant.

The follow-up outpatient clinic appointment can by conducted by the PI, Co-I, RN or RP. As well as discussing details of the MACRO trial, the following elements of the consent process should be outlined:

1. A trial-specific ECG must be carried out to confirm eligibility and consent is required for this.
2. A pregnancy test must be undertaken by females of childbearing potential and consent is required for this.
3. The patient will be requested to consent to providing their email address and mobile phone number to enable the electronic completion of PROM questionnaires and resource-use diaries. If the patient does not consent to completing the documentation electronically, they will be asked instead to provide their home address and telephone number so that the MACRO trial office can send the PROM questionnaires and resource-use diaries by post.
4. The participant has the option to consent to being contacted in the future about the MACRO trial or participating in other research studies (e.g. by giving tissue, blood or mucus samples).
5. The participant has the option to consent to an interview with a qualitative researcher as part of the mixed-methods evaluation.
6. The participant has the option to consent to being followed up annually for up to 5 years as part of the larger MACRO programme of work. Participants participating in the long-term follow-up will be requested to complete the SNOT-22 and EQ-5D-5 L validated questionnaires, and a short questionnaire online (a paper version is also available).

A trained RN or RP, the local PI, the Co-I or another appropriately qualified member of the research team will obtain informed consent. The person taking informed consent will be trained on good clinical practice and will be suitably qualified and experienced. They will have been delegated this duty by the chief investigator or PI, which will be recorded on the staff signature and delegation log. The investigator or designee will explain to the patient that they are under no obligation to enter the trial and that they can withdraw at any time during the trial without providing a reason. No clinical trial procedures will be conducted prior to the participant giving consent by signing the informed consent form. Consent will not denote enrolment into the trial.

A copy of the signed informed consent form will be given to the participant. The original signed form will be retained in the trial file at the site and a copy placed in the medical notes. The PIS and informed consent form will be reviewed and updated if necessary during the trial (e.g. where new safety information becomes available) and participants will be re-consented as appropriate.

#### Declaration of interests

The members of the research team have no financial or other competing interests.

#### Ancillary and post-trial care

At the completion of the trial, the patient will receive the normal clinical care provided by the primary-care physician and treating ENT surgeon.

#### Dissemination policy

As part of the wider body of work of the MACRO programme, we anticipate dissemination through traditional channels, including conference presentations, abstracts and open-access peer-reviewed publications. These will be targeted at both specialist and generalist groups, facilitated by the roles held by the members of our collaboration in relevant professional societies and guideline-producing bodies. However, beyond informal and specialist publications, we also plan to use wider communication channels to allow larger-scale dissemination to the general public. When our public and patient involvement panel has facilitated a patient-friendly and comprehensible summary of the study findings, these will be provided to all study participants by mail and to the wider public via a study website. We will maximise non-professional dissemination through a number of multimedia outlets, including targeted mailshots, press releases to medical and general journalists, health information websites and communications media, evidence-based interest groups, conference presentations, social media, patient interest groups and medical charities, such as Fifth Sense and Asthma UK. We anticipate the results may lead to the production of new guidelines, which will be made available through websites such as those of ENT-UK and the Royal College of Surgeons.

## Discussion

The MACRO trial is part of the MACRO programme funded by the National Institute of Health Research Programme Grants for Applied Research. The overarching programme is run by a programme management group. As listed in Additional file 1: Appendix 1, the trial is managed by the TMG with independent oversight from the PSC and the DSMC. Day-to-day trial administration is the responsibility of the trial manager in liaison with the chief investigators. The trial manager is supported by colleagues at SITU, which is part of the clinical trials unit of the UK Clinical Research Collaboration, OCTRU (Additional file 1: Appendix 1). The sites for the trial were selected based on volume of cases and prior engagement of the PIs in the research group at the British Rhinological Society, and also to achieve a mix of teaching hospitals and district general hospitals over a wide geographical area.

The effectiveness of antibiotics in the treatment CRS will be established through the trial, which may help to reduce unnecessary usage and potential morbidity. If ESS is shown to be clinically effective and cost-effective, the trial may encourage earlier intervention. In contrast, if it is shown to be ineffective, then there should be a significant reduction in surgery rates. The trial results will feed into the other components of the MACRO research programme to establish best practice for the management of adults with CRS and design the ideal patient pathway across primary and secondary care.

### Trial status

The current protocol is version 3.0, dated 31 August 2018 (sponsor protocol number 14/0644). The trial is currently in the pilot phase, having officially commenced in January 2019 and is due to complete in 2022 (Additional file 2).

## Additional files

Additional file 1: **Appendix 1.** Trial registration, protocol version, funding details, roles and responsibilities. **Appendix 2.** Schedule of enrolment, interventions, and assessments (Spirit guidelines figure). (DOCX 1089 kb)

Additional file 2: SPIRIT 2013 Checklist: Recommended items to address in a clinical trial protocol and related documents. (DOCX 53 kb)
