# Supplementary material for: Clarithromycin and endoscopic sinus surgery for adults with chronic rhinosinusitis with and without nasal polyps: study protocol for the MACRO randomised controlled trial
Source: Trials. 2019 Apr 29;20:246. doi: 10.1186/s13063-019-3314-7 (PMC6489242; doi:10.1186/s13063-019-3314-7)

A Randomised Controlled Trial of Clarithromycin and Endoscopic Sinus Surgery for Adults with Chronic Rhinosinusitis with and without nasal polyps: study protocol for the MACRO randomised controlled trial

**Authors:** Carl Philpott, Steffi le Conte, David Beard, Jonathan Cook, William Sones, Steve Morris, Caroline S Clarke, Mike Thomas, Paul Little, Jane Vennik, Valerie Lund, Helen Blackshaw, Anne Schilder, Stephen Durham, Spiros Denaxas, James Carpenter, James Boardman, Claire Hopkins

**Affiliations:**

1. Prof Carl Philpott, Norwich Medical School, University of East Anglia, Norwich, UK; ENT Department, James Paget University Hospital NHS Foundation Trust, Great Yarmouth, UK
2. Miss Steffi le Conte, SITU, University of Oxford, Oxford, UK
3. Prof David Beard, SITU, University of Oxford, Oxford, UK
4. Prof Jonathan Cook, SITU, University of Oxford, Oxford, UK
5. Dr William Sones, SITU, University of Oxford, Oxford, UK
6. Dr Caroline S Clarke, Research Department of Primary Care and Population Health, University College London, London, UK
7. Prof Stephen Morris, Department of Applied Health Research, University College London, London, UK
8. Prof Mike Thomas, University of Southampton, Southampton, UK
9. Prof Paul Little, University of Southampton, Southampton, UK
10. Dr Jane Vennik, University of Southampton, Southampton, UK
11. Prof Valerie Lund, UCL Ear Institute, University College London, London, UK
12. Dr Helen Blackshaw, evidENT, UCL Ear Institute, University College London, London, UK
13. Prof Anne Schilder, eviDENT, UCL Ear Institute, University College London, London, UK
14. Prof Stephen Durham, Faculty of Medicine, National Heart & Lung Institute, Imperial College London, UK
15. Dr Spiros Denaxas, Institute of Health Informatics, University College London, London, UK
16. Prof James Carpenter, London School of Hygiene and Tropical Medicine, University College London, London, UK
17. Mr James Boardman, Fifth Sense, UK
18. Prof Claire Hopkins, ENT Department, Guy’s and St Thomas’ NHS Foundation Trust, London, UK

On behalf of the MACRO programme team.

Corresponding author: Carl Philpott [C.Philpott@uea.ac.uk](mailto:C.Philpott@uea.ac.uk)

Sponsor contact: Keith Boland [k.boland@ucl.ac.uk](mailto:k.boland@ucl.ac.uk)

This paper has been written in accordance with the SPIRIT statement for trial protocols.

Protocol Version V3.0 dated 31st August 2018; Sponsor protocol number: 14/0644

**Funder:** National Institute of Health Research (NIHR): Programme Grant for Applied Research Stream (PGfAR), Reference Number: RP-PG-0614-20011

[clairehopkins@yahoo.com](mailto:clairehopkins@yahoo.com); macrotrial@nds.ox.ac.uk; a.schilder@ucl.ac.uk; [v.lund@ucl.ac.uk](mailto:v.lund@ucl.ac.uk); h.blackshaw@ucl.ac.uk; d.m.thomas@soton.ac.uk; psl3@soton.ac.uk; steve.morris@ucl.ac.uk; caroline.clarke@ucl.ac.uk; j.carpenter@ucl.ac.uk; s.denaxas@ucl.ac.uk; wychall44@gmail.com; william.sones@ndorms.ox.ac.uk; david.beard@ndorms.ox.ac.uk; jonathan.cook@ndorms.ox.ac.uk; s.durham@imperial.ac.uk; j.vennik@soton.ac.uk

# Abstract

**Background:** Chronic Rhinosinusitis (CRS) represents a common source of ill health; 11% of UK adults reported CRS symptoms in a worldwide population study. Guidelines are conflicting regarding whether antibiotics should be included in primary medical management, reflecting the lack of evidence seen from systematic reviews. Insufficient evidence to define the role of surgery contributes to a 5-fold variation in UK intervention rates. The objective of this trial is to establish the comparative effectiveness of endoscopic sinus surgery (ESS) or a prolonged course of antibiotics (clarithromycin) in adult patients with CRS in terms of symptomatic improvement and costs to the National Health Service (NHS) compared with standard medical care (intranasal medication) and each other at six months.

**Methods:** A 3-arm parallel group trial will be conducted where patients who remain symptomatic after receiving appropriate medical therapy (either in primary or secondary care) will be randomised to receive either **intranasal medication plus endoscopic sinus surgery**, **intranasal medication plus clarithromycin (250mg)** or **intranasal medication plus placebo**. Intranasal medication (current standard medical care) is defined as intranasal corticosteroids (INCS) spray or drops and saline irrigations. The primary outcome measure is the disease-specific health related quality of life SNOT-22 questionnaire. The study sample size is 600. Principal analyses will be according to the randomised groups irrespective of compliance. The trial will be conducted through at least 16 secondary/tertiary care centres with an internal pilot at 6 sites for 6 months.

**Discussion:** The potential cardiovascular side-effects of macrolide antibiotics have been recently highlighted. The effectiveness of antibiotics will be established through the trial, reducing unnecessary usage and potential morbidity. With respect to ESS, if it is shown to be clinically and cost effective, the trial may encourage earlier intervention. In contrast, if shown to be ineffective, this should achieve significant reductions in surgery rates. The trial results will feed into the other components of the MACRO research programme to establish best practice for the management of adults with Chronic Rhinosinusitis (CRS) and design the ideal patient pathway across primary and secondary care.

**Trial registration:** ISRCTN no: 36962030 (17^th^ October 2018)

**Keywords:** chronic rhinosinusitis, endoscopic sinus surgery, clarithromycin, randomised controlled trial

# Introduction

## Background and rationale

### The problem being addressed

Chronic Rhinosinusitis (CRS) represents a common source of ill health; 11% of UK adults reported CRS symptoms in a worldwide population study^[1].^ Symptoms, including nasal obstruction, nasal discharge, facial pain, anosmia and sleep disturbance, have a major impact on quality of life (QoL), reportedly greater in several domains of the SF-36 than angina or chronic respiratory disease^[2]^. Acute exacerbations, inadequate symptom control and respiratory disease exacerbation are common. Complications are rare but may include visual impairment and intracranial infection. Longitudinal primary care electronic health records from the Clinical Practice Research Datalink (CPRD), a nationally representative subset of primary care data across consenting GP practices, show that 1% of UK adults receive treatment for CRS from their GP each year, with a median of four GP visits; they receive multiple medications with 91% receiving an antibiotic prescription^[3].^ Secondary care electronic health records from Hospital Episode Statistics (HES), England’s national administrative billing dataset for hospitals, for 2012-13, show that approximately 40,000 sinus operations were performed in England and Wales, in addition to an estimated 120,000 outpatient consultations^[4]^. A worldwide study demonstrated that one in three CRS patients in primary care have poorly controlled symptoms^[5]^. The socio-economic cost of CRS is significant with 57% of patients reporting absenteeism and 28% experiencing associated anxiety and depression^[6] [7]^. US Businesses report CRS as one of their top 10 illnesses causing work absence^[8]^.

### Need and rationale for research in this area

The European Position Paper on Rhinosinusitis and Nasal Polyps (EPOS 2012) published treatment guidelines and a research strategy for CRS, emphasising where limited evidence restricts care^[9]^. Key elements of this strategy are addressed herein, including the paucity of RCTs in rhinosinusitis treatments. In the UK, there are no specific national treatment guidelines and uptake of EPOS 2012 is variable^[10]^. A recent ENT-UK commissioning guideline^[11]^ acknowledges the lack of high quality trials and although it does not recommend routine antibiotic use for CRS in primary care, GPs often prescribe repeated courses^[12]^ which may cause resistance. There is growing interest in immune-modulating and anti-inflammatory effects of macrolide antibiotics in chronic airway inflammatory disease, with low-dose long term macrolide being prescribed for its immune response and not primarily as an anti-bacterial agent^[13]^. Longer-term antibiotic use in secondary care has a low-grade recommendation reflecting conflicting evidence from two RCTs^[14, 15]^ resulting in a call for further trials^[16-18]^.

First line therapy is often delivered in primary care, and consists of initial treatment with intranasal corticosteroids, short courses of antibiotics and / or saline rinses. At least one in three CRS patients attending ENT clinics are considered to have failed this initial treatment, hereafter called appropriate medical therapy (AMT) and are considered for endoscopic sinus surgery (ESS)^[19, 20]^. Insufficient evidence to define the role of surgery contributes to a 5-fold variation in intervention rates across England, by Clinical Commissioning Group (CCG)^[11]^. Symptom duration before surgery varies from under one to over 10 years^[21, 22]^. If surgery is less effective than AMT, patients may be exposed to unnecessary risks and morbidity. If surgery is more effective, current delays reflect suboptimal patient care. Such uncertainty resulted in inclusion of ESS in the NICE Database of Treatment Uncertainties (DUETS)^[23]^.

As the EPOS 2012 guidelines recommend AMT prior to surgery, any trial must ensure patients have failed to respond to an adequate attempt at treatment using AMT. Intranasal corticosteroids and saline irrigation, for which there are strong recommendations for use, based on high-quality level 1 evidence, should be included in AMT. Guidelines are conflicting regarding whether antibiotics should be included in primary medical management, reflecting the lack of evidence above. Our trial uses a pragmatic approach where eligibility for surgery is based on a shared decision between the Principal Investigator (PI) and patient, after initial treatment with AMT, in keeping with current guidelines ^[9, 24, 25]^.

### Past and current research

Cochrane reviews have recently been completed covering the treatment options being assessed in this trial^[26-31]^ supporting the use of intranasal corticosteroids and saline irrigations as standard treatment but demonstrating the need for more trials with antibiotics. An HTA commissioned systematic review of ESS identified the need for high-quality studies comparing surgery with medical treatment^[32, 33]^. Updated reviews of the literature and trial registries have not identified new studies. One potentially relevant trial reported by DUETS was never started. Two 2014 Cochrane systematic reviews of medical and surgical management concluded that further studies are urgently needed^[34, 35]^. There is little ongoing research that overlaps with the MACRO programme. Two relevant antibiotic trials are currently recruiting. A trial aiming to determine the optimum duration of longer-term antibiotics in patients with CRS ^[36]^ compares the effectiveness of 3 with 6 weeks of azithromycin, but fails to include a placebo arm. A commercially sponsored study compares azithromycin with placebo in patients with persistent symptoms after ESS ^[37]^, but for many patients the aim is to avoid primary surgery^[38]^, and therefore a trial to determine the effectiveness of antibiotics compared with ESS is essential. Finally, there is an RCT comparing ESS with topical medical therapy versus topical medical therapy alone for the Chronic Rhinosinusitis with nasal polyps (CRSwNPs) subgroup only, underway in the Netherlands^[39]^.

### Importance of the research

Despite the burden of CRS, UK health-economic evaluations are lacking. An American model demonstrates a high economic burden to patients, healthcare systems and society ^[40, 41]^; in 2011 CRS cost the US healthcare system $8.6 billion with significant direct and indirect costs. Antibiotic resistance is considered one of the most significant threats to patients’ safety in Europe^[42]^. Evaluating effectiveness of antibiotics and promoting appropriate usage is integral to the UK 5-year antimicrobial resistance strategy^[43]^. Given the high prevalence of CRS and the variability in antibiotic prescribing, this may represent a public health danger through selective pressure on bacteria and antibiotic resistance. In addition, the potential cardiovascular side-effects of macrolide antibiotics have been recently highlighted^[44]^. The effectiveness of antibiotics must be urgently established, reducing unnecessary usage and potential morbidity. International guidelines suggest that patients should receive ‘maximum medical therapy’ (which usually includes nasal (and possibly oral) steroids, antibiotic treatment and regular nasal douching) prior to surgical intervention, however use and duration of antibiotic use is very variable. 91% of CRS patients currently receive an antibiotic prescription prior to referral; recently some CCGs have insisted on a 3 month trial of macrolide antibiotics prior to secondary care referral^[45]^; yet evidence to support this recommendation is lacking.

There is both wide variation in surgical practice, and very high rates of revision surgery^[46] [47]^. There is a risk patients may undergo surgery and its attendant complications without due reason. In contrast recent changes to commissioning, restricting access to sinus surgery, may deny beneficial treatment to patients. The risk is that limited evidence for effectiveness is assumed to convey non-effectiveness, leading to decommissioning of an effective treatment by CCGs, with some already deviating widely from NICE commissioning guidelines^[48]^. It is essential to rigorously assess the effectiveness and cost-effectiveness of surgery to enable robust decision-making from patient, health service and societal perspectives. In non-randomised studies, surgery achieves significant improvement in health-related QOL and reductions in ongoing healthcare utilisation. Pre- and post-trial health economic modelling will ensure the trial design reflects current pathways. If ESS is clinically and cost effective, the trial may encourage earlier intervention. In contrast, if shown to be ineffective, this should achieve significant reductions in surgery rates.

The MACRO trial forms part of a larger programme of work, the overarching aim of which is to address the major deficiencies in the evidence base for CRS management, establish best practice for the management of adults with (CRS) and design the ideal patient pathway across primary and secondary care.

## Objectives

### Primary objective

To establish the comparative effectiveness of a prolonged course of antibiotics (clarithromycin) or endoscopic sinus surgery (ESS) in adult patients with CRS in terms of symptomatic improvement and costs to the NHS, compared with standard medical (intranasal medication) care and each other at six months.

### Secondary objectives

- Measure clinical effectiveness using subjective self-reporting ratings and objective clinical measures
- Compare the clinical effectiveness according to phenotype – CRS with and without polyps (CRSwNPs/CRSsNPs)
- Record the incidence and details of adverse events in all treatment arms related to the trial medication or surgery intervention
- Establish the cost effectiveness and cost utility of each arm relative to the others over the 6-month trial duration
- Embed a mixed methods evaluation into the main trial to identify factors and processes necessary for implementation of trial findings.

### Internal recruitment pilot phase objectives

- To randomise 72 patients at six pilot sites within six months. Recruitment will be deemed successful if ≥75% of expected, i.e. 54 patients, are recruited.
- Undertake an embedded qualitative study (the MACRO Conversation Study) during the pilot phase to identify recruitment challenges and optimize recruitment during the main trial phase.

## Trial design

### Overall design

The MACRO trial is a multi-centre 3-arm placebo controlled parallel group RCT that aims to randomise 600 adult patients with CRS. Patients who remain symptomatic after receiving AMT as deemed suitable by the local PI or Co-I (either in primary or secondary care) and who are considered suitable candidates for further treatment (including surgery) will be randomised on a 1:1:1 basis to receive:

- **ESS: Intranasal medication** plus **ESS** within 6 weeks of randomisation
- **ANTIBIOTIC: Intranasal medication** plus an initial **2 week** course of **clarithromycin 250mg** **capsules twice daily** followed by a **10 week** course of **clarithromycin 250mg capsules once daily**
- **PLACEBO: Intranasal medication** plus an initial **2 week** course of **placebo** **capsules twice daily** followed by a **10 week** course of **placebo capsules once daily**

**Intranasal medication** (current standard medical care) is defined as intranasal corticosteroids (INCS) spray or drops and saline irrigations as per local formulary guidelines and saline rinses will be provided by the research nurse. These are non-investigational medicinal products (NIMPs) (see section 6.3)

### Internal Recruitment Pilot Phase

At least three sites should be open for the six-month internal recruitment pilot phase to begin; six sites in total will be included. An embedded qualitative study (the MACRO Conversation study) will be conducted as part of the pilot phase to identify any challenges to recruitment. The qualitative work will involve audio-recording of recruitment consultations, and in-depth interviews with trial staff and patients who have been approached about participating in the MACRO trial. Outcomes of the qualitative work will be used to inform any changes to the design or conduct of the study, and to identify recruitment strategies or training needs to optimise recruitment for the main trial phase. If recruitment is lower than expected, we will propose a plan to increase recruitment based on the findings from the MACRO Conversation Study and communicate regularly with the funder.

### Main Trial Phase

On the basis of the pilot phase being successful, further pre-identified sites will be opened, and all sites will recruit the remaining participants required to achieve a sample size of 600 within the funded timescale of the MACRO trial (estimated total trial duration is 52 months). In terms of follow up, both work performed by the research team ^[47, 49]^ and independent researchers ^[50]^ has shown that improvements in QOL remain stable between 6 and 60 months. Soler concluded that for clinical trials incorporating QOL outcomes, 6 months is considered to be an appropriate primary end-point ^[50]^ and therefore 6 months is the primary end-point for this study. Subject to additional funding, trial follow-up is proposed for a further 40-60 months after the initial 52 months’ trial duration, to enable completion of long-term follow-up for all participants who have given their consent (up to 5 years).

# Methods:

## Study setting

The trial will be conducted through at least 16 secondary/tertiary care centres around the UK with a dedicated Consultant Rhinologist as the Principal Investigator at each site. Details of sites can be found on the trial website: <https://workstream2.themacroprogramme.org.uk/>

## PARTICIPANTS

### Eligibility of trial participants

Adult patients with CRS, where symptom control has not been achieved following previous appropriate medical therapy (AMT) and who are considered suitable candidates for further treatment (including surgery), will be assessed for eligibility. Eligibility must be assessed by a medically qualified doctor and only personnel formally delegated by the PI to assess eligibility on the trial-specific delegation log may perform this task.

### Trial participant inclusion criteria

- Adults aged 18 and over with a diagnosis of CRS according to European guidelines:
- A minimum of 12 weeks’ history of inflammation of the nose and paranasal sinuses characterised by two or more symptoms, one of which should be either nasal blockage/obstruction/ congestion or nasal discharge (anterior/posterior nasal drip):
  - - ± facial pain/pressure
    - ± reduction or loss of smell
- Nasal endoscopy (within last 3 months) to determine CRS diagnosis and phenotype (CRSwNPs or CRSsNPs)
- Non-contrast CT scan (within last 12 months) to determine Lund-Mackay score and confirm suitability for ESS
- Moderate/severe symptoms; SNOT-22 score ≥ 20 (within last 3 months)
- Symptom control not achieved following previous AMT, as deemed by the local Principal Investigator (PI) or Co-Investigator (Co-I), and therefore considered eligible for ESS
- An understanding of the English language sufficient to understand written and verbal information about the trial, its consent process and the study questionnaires

### Trial participant exclusion criteria

- Lund-Mackay non-contrast CT scan score < 4
- Macrolide antibiotic treatment for > 3 continuous weeks’ duration within the last 12 months
- ESS in previous 6 months or visible, open sinus cavities from previous surgery
- Maintenance oral steroids or biologics within last 3 months
- Rare/complex sinus conditions:
- CRS secondary to systemic disease – e.g. ciliary dyskinesias, granulomatous diseases
- Suspected malignancy
- Allergic fungal rhinosinusitis confirmed or suspected on CT imaging (expansion and mixed density opacification) necessitating immediate surgery
- Severe asthma (high doses of inhaled steroids i.e. >1.5mg per day)
- Females who are pregnant or breastfeeding, females of reproductive potential not prepared to use a reliable means of contraception (e.g. hormonal contraceptive patch, intrauterine device, physical barrier or abstinence, if preferred and usual lifestyle of the patient) at trial entry or those females wanting to start a family during the initial 3 months of the trial
- Known immunodeficiency states including HIV and selective and multiple antibody deficiency states
- Severe septal deviation preventing endoscopic examination
- Contraindications to surgery (significant medical co-morbidity)
- Any absolute contraindications to clarithromycin (risk factors to be assessed at screening include history of ischaemic heart disease, prolonged QT interval on ECG, diabetes and age over 65 or any medications known to interact with clarithromycin unless these can be discontinued during the 3 months of clarithromycin/placebo treatment
- Known allergies to the IMP and excipients of IMP and placebo
- Inability to give consent (significant cognitive impairment or language issues), or to understand and comply with trial instructions
- Participation in another Randomized Clinical Trial in the past 4 months

### Pre-trial routine assessments to assess eligibility

All patients seen in clinic by the research team will undergo a routine nasal endoscopy to determine CRS diagnosis and phenotype (CRSwNPs or CRSsNPs). The patients will also be asked to complete a routine SNOT-22 validated Patient Reported Outcome Measures (PROM) questionnaire to determine the severity of symptoms (score of ≥20 needed to be assessed for eligibility). Previous medical therapy will be discussed, and if symptoms have persisted following AMT (as deemed by the PI/Co-I) and the patient is considered suitable for further treatment (including surgery), a routine CT scan will be requested, in order to confirm CRS and determine the Lund-Mackay score. The screening, consent and randomisation flow diagram is shown in Appendix 2.

## Interventions

### Name and description of IMP(s)

UK licensed clarithromycin standard release 250mg tablets will be over-encapsulated and provided in two bottles with a blinded annex 13 compliant label. The first bottle will contain the induction dose (one capsule to be taken orally twice daily for 2 weeks) and the second bottle will contain the remaining capsules, to be taken orally once a day for a further 10 weeks. The placebo capsules will also be provided in matching bottles with randomised, blinded labels.

### Choice of antibiotic for the trial

With respect to the choice of antibiotic, we have selected the macrolide class and specifically clarithromycin for a number of specific reasons:

- It has both anti-inflammatory and antimicrobial properties as demonstrated in other respiratory tract disorders ^[51-53]^. At a dose of clarithromycin 250 mg orally twice daily, the peak tissue levels 4 hours after administration have been shown to be 8.32 mg/kg±2.57 in nasal mucosa, with the drug characteristic being therapeutic serum concentrations and high tissue concentrations^[54]^. As a result, clarithromycin exhibits its immunomodulatory effects through inhibition of neutrophilic inflammation and macrophage activation^[55]^.
- It should provide good coverage of typical CRS flora^[56]^. A dose of 250mg twice daily is effective for treatment of respiratory tract organisms including Moraxella catarrhalis, Streptococcus pneumoniae, Staphylococcus aureus or Haemophilus inﬂuenzae (Minimum inhibitory concentration for 90th percentile of the isolates = 0.064 mg/L)^[57]^.
- It is currently recommended (Grade C recommendation) by EPOS and the ENT UK rhinosinusitis commissioning guidelines in selected patients with CRS ^[9, 58]^
- Although EPOS states that use should be restricted to patients who have confirmed CRS on endoscopic examination, it is increasingly being recommended that GPs prescribe a prolonged course to patients without confirmatory endoscopy in primary care; however, in the MACRO Programme Workstream 1, health informatics data showed little evidence of courses greater than 3 weeks being prescribed in primary care.
- There are two RCTs that have conflicting results for the efficacy of macrolides^[14, 15]^ necessitating a trial that can differentiate according to phenotype.
- Previous studies suggest a longer course of medication is better^[59, 60]^, although in practice both duration and compliance varies, but we propose to adhere to a 12-week course of clarithromycin within this trial, in order to adhere to realistic timelines and recruitment targets for this study.
- Systematic reviews have highlighted that the overall quality of evidence from previous studies was low due to limitations in trial design and state that a firm conclusion on the effectiveness of macrolides in CRS could not be reached ^[26, 61]^.
- There have been a number of publications raising concerns about cardiac toxicity with erythromycin in patients with a prolonged QT interval ^[62, 63]^. We have selected clarithromycin due to poorer tolerability with erythromycin, poor efficacy of azithromycin in the previous RCT and lack of availability of roxithromycin in the UK
- Aside from the issues above, clarithromycin is readily available and has a reasonable side-effect profile (as seen in our feasibility study) ^[64]^

### Name and description of each NIMP

Intranasal corticosteroids (INCS) spray or drops will be prescribed for all participants to use throughout the duration of the trial, as per local formulary practice. Saline irrigation packs will be provided by NeilMed® Pharmaceuticals. Both concomitant medications are UK licensed medicines and used within their indication. These drugs are considered to be non-investigational medicinal products (NIMPs) in this trial. Host sites are responsible for maintaining a system that allows adequate reconstruction of NIMP movements; these will be recorded on the case report forms*.*

### Concomitant medication

In the case of acute exacerbations of the condition, participants may receive appropriate additional medical treatment as decided by the ENT surgeon or GP. This can include oral steroids or full dose broad spectrum or culture-directed antibiotics and details of these concomitant medications will be captured in the patient reported resource use diaries (to be completed at baseline and at 3 and 6 months). A list of medicinal products contraindicated in the use of IMP is available on request from the trial manager at [macrotrial@nds.ox.ac.uk](mailto:macrotrial@nds.ox.ac.uk) .

### Endoscopic Sinus Surgery (ESS)

ESS will be performed by consultant rhinologists according to the techniques described by Stammberger, Lund and Kennedy, with surgery proceeding in a stepwise fashion through polypectomy (where present), uncinectomy, middle meatal antrostomy, ethmoidectomy, with additional frontal and sphenoid surgery in selected participants. Extent of surgery to be performed will be decided at an individual participant level but recorded by the operating surgeon as part of the CRF (having first been documented in the medical notes). Instrumentation will not be standardised between centres. Surgery can be undertaken by a registrar under consultant supervision. As this is a pragmatic trial, there will be no specific further standardisation of the ESS episode at each site.

### Treatment Schedule

See also overall trial design above. Participants will receive a prescription to take to the pharmacy for the INCS spray/drops and will receive a 3-month supply to last them until their next scheduled visit. Participants will also be supplied with sinus irrigation bottles and sachets provided by NeilMed Pharmaceuticals Inc. (P.O. Box 2853. Coulsdon, Surrey, CR5 2WN, United Kingdom). Each site will have a designated pharmacy lead for the trial, who will be informed by Oxford SITU as to the randomisation outcome and the requirement for clarithromycin or placebo medication.

### If the participant does not receive surgery within 6 weeks of randomisation, this is not considered a protocol non-compliance. The date of surgery will first be captured in the patients’ medical notes and then transcribed onto the treatment CRF from the medical records. Dose Modifications

There are no dose modifications in the MACRO trial. Should a patient be unable to tolerate the dose of medication they will discontinue treatment but be requested to remain in the trial; please refer to sub-section ‘Stopping trial treatment’. In terms of the dosage of clarithromycin, this is in line with an open-label study in the field^[65]^ and 250mg is a readily available dose. The initial regimen is for two weeks as this is considered to be therapeutic in terms of an antibiotic effect; participants will then be requested to take clarithromycin 250mg od (once a day) for a further 10 weeks as the key purpose of the medication in the MACRO Trial is immunomodulatory^[53]^; only a lower dose is required for this effect to be exerted and takes into consideration recent concerns around cardiovascular morbidity that were addressed by Workstream 1 as part of our trial consensus process.

### Assessment of IMP/NIMP compliance

Compliance with medical treatment will be recorded in the weekly compliance diaries completed by the participants.

### Stopping trial treatment

Participants allocated either to the active IMP or placebo will be requested to carry a card with them at all times, outlining that they are participating in the MACRO trial.

- Should any suspected, serious interactions (or serious adverse events that are deemed related to the study medication) occur in those participants randomised to either clarithromycin or placebo, the attending clinician, PI or Co-I should request the participant to stop taking the trial medication immediately, and inform the MACRO Trial Office using the secure SITU e-mail address ([situ.oxford@nhs.net](mailto:situ.oxford@nhs.net)) from an NHS e-mail account.
- If the participant stops taking their trial medication due to unwanted side effects, the participant must inform the PI/Co-I or Research Nurse (RN)/Research Practitioner (RP) as soon as possible. The PI/Co-I or RN/RP should then contact the trial office using the secure e-mail [situ.oxford@nhs.net](mailto:situ.oxford@nhs.net).

In both cases, the PI/Co-I or RN/RP should first document this in the medical notes, and then complete a ‘Change of Status’ CRF detailing that the participant has stopped taking their trial medication and the reason for stopping. The CRF should be sent to the MACRO trial office. The participant will be requested to remain in the trial and continue to undertake all follow-up activities, as per this protocol (even if they are unblinded to their study treatment). The patient should be requested to return any unused trial medication at their next follow-up visit.

If the participant is allocated to ESS and the treatment does not go ahead, or is changed part way through (for clinical reasons), this should first be documented in the medical notes, and a ‘Change of Status’ CRF should be completed. The CRF should be sent to the MACRO trial office. The participant will be requested to remain in the trial and continue to undertake all follow-up activities, as per this protocol.

A ‘Change of Status’ CRF should be completed by the PI/Co-I or RN/RP and sent to the MACRO trial office if the patient’s treatment deviated from the allocated intervention. The participant will be requested to remain in the trial and continue to undertake all follow-up activities, as per this protocol.

## Outcomes

| **Primary** | Disease-specific health related quality of life (HRQoL) using the SNOT-22 at six months |
| --- | --- |
| **Secondary** | - Endoscopic score (Lund-Kennedy Score) - Grade of polyps (0 – 3, Lildholdt Score) - Health-related quality of life and quality-adjusted life-years (QALYs), measured by the SF-12v2 and EQ-5D-5L questionnaires - Need for additional treatment (e.g. oral steroids, antibiotics etc.) - Olfactory function measured using Sniffin’ Sticks - Upper and lower respiratory function, measured using peak expiratory flow rate, peak nasal inspiratory flow rate - Asthma Control Test (ACT) (participants with asthma only) - Adverse events of treatment - Healthcare resource use, including medications and visits to primary and secondary care, recorded using patient questionnaires - Days of work missed, recorded using patient questionnaires - Overall cost and incremental cost per quality-adjusted life-year gained, from the cost perspective of the NHS and PSS, calculated using quality of life scores - Budget impact of treatment |

## Participant timeline

See details of treatment schedule above and participant flow chart in Figure 1. All participants will be enrolled in the trial for a period of six months from randomisation. Appendix 3 shows the visit schedules.

### Visit schedule, baseline and follow-up assessments

Once informed consent has been obtained and the ECG and pregnancy test (where applicable) have confirmed the participant’s eligibility, a comprehensive baseline assessment will be undertaken:

- Clinical assessments:-
  - Peak Expiratory Flow Measurement
  - Peak Nasal Inspiratory Flow Measurement
  - Sniffin' Sticks olfactory test
  - Skin prick allergy test (or RAST inhalant screen)*
- Blood tests to include full blood count and total IgE count (results from blood tests taken within last 6 months are acceptable, however the participant must not have taken prednisolone or other oral steroids within 6 weeks preceding blood test)
- Eligibility confirmation
- Participant demographics and medical history

*Minimum allergens to be tested: HDM, mixed grass, mixed tree, mixed mould, dog and cat. Specific allergens are acceptable in place of mixed tree/grass/mould.

Results of these assessments will be recorded in the baseline CRF (and medical notes if clinically important) along with data from the routine care pre-screening assessments:-

- Lund Mackay score from the CT scan
- Lund-Kennedy score from the endoscopy
- Lildholdt polyp grade from the endoscopy
- SNOT-22 score

Participants will be requested to complete the following baseline disease specific and generic PROMs online: SNOT-22, SF-12v2 and EQ-5D-5L questionnaires during the baseline clinic visit, as well as asthma control test (ACT), if known asthmatic. Participants will also be requested to complete an initial online baseline resource use questionnaire, which will ask participants about their use of health care services over the last 3 months before joining the trial. These questionnaires will be completed online by the participant, but during the baseline visit so that the RN/RP is on hand to answer any questions. If any site has difficulties with doing these questionnaires online, a paper version is also available. Following the initial baseline assessment, all participants in all treatment arms will be followed up in clinic at three and six months (+/- 7 days) post-randomisation. The following measurements will be undertaken and recorded on a trial specific follow-up CRF:-

- Nasal endoscopy
- Peak Expiratory Flow Measurement
- Peak Nasal Inspiratory Flow Measurement
- Sniffin' Sticks olfactory test

Women of childbearing potential will not be requested to undertake another pregnancy test at their 3 or 6 month post-randomisation clinic visit. However, the site should reiterate to the woman that if pregnancy occurs, a member of the local site staff should be informed immediately, and a Pregnancy Reporting Form should be completed and sent to the central MACRO Office.

Participants will be contacted separately by the MACRO trial office and requested to electronically complete the SNOT-22, EQ-5D-5L and SF-12v2 PROM questionnaires at 6 weeks, 3 months and 6 months post randomisation. The participant will also be asked to complete an ACT (if known asthmatic) at 3 and 6 months. Those participants who state at recruitment that they cannot, or do not wish to complete these questionnaires online will be sent the paper version by the MACRO trial office and requested to complete and return the questionnaires in a pre-paid envelope.

Participants will also be asked to complete a weekly questionnaire (recording compliance with intranasal and placebo/clarithromycin trial-specific medication usage) and a separate Health Economics resource use questionnaire at baseline, 3 and 6 months (recording other medication and healthcare resource use, and details of time off work and other costs) in order to evaluate:-

- Need for additional treatment (e.g. oral steroids, antibiotics etc.)
- Adverse effects of treatment
- Healthcare visits to primary and secondary care
- Days of work missed
- Cost and cost-effectiveness from the perspective of the NHS and personal social services (PSS)
- Compliance with the trial medication

A schedule of all trial assessments and procedures is set out in Appendix 3. Samples will be taken for a full blood count and total IgE and will be carried out at local laboratories.

## Sample size

The trial will recruit 600 participants. Participants being recruited from 17 centres (therefore 17 ESS surgeons) is considered a realistic assumption for the following sample size calculations.. Sample size justification is based upon achieving at least 80% statistical power at the 2-sided 5% significance level. No adjustment for multiple comparisons has been made as each of the treatment comparisons are distinct. The minimum clinically important difference (MCID) has been estimated to be about 8.9 points based upon an anchor study^[66]^. A 10-point difference in SNOT-22, (0.5SD, Cohen’s effect size assuming an SD of 20) is often considered a medium sized effect size and an important difference for this type of outcomes. A previous study suggests that a larger effect for surgery against alternative treatment is plausible, as large as 13.8^31^. Using target differences of and 8.9 and 10 (and SD of 20) would require and 107 and 90 per group (331 and 270 overall) to achieve 90% statistical power at the 2-sided 5% significance level. Offsetting this is the possibility of clustering within the surgical arm which affects pairwise comparisons involving surgery, and the need to perform subgroup analysis for CRSwNPs versus CRSsNPs participants. Allowing for clustering (ICC of 0.05 and 17 clusters of equal size) in the surgical group would led to 102 per group (306 overall) for a target difference of 10 points for 90% power. For this trial size and using a target difference of 8.9 but an otherwise identical calculation there would be 80% power. To enable analyses of treatment by polyp presence subgroup interaction to be analysed in secondary analyses, the overall sample size was inflated to 600 (after allowing for 10% missing data). This size of study would allow us to detect a difference of 8.9 and 10 (SD of 20) to be detected in all CRS patients with >90% power after adjusting for clustering for the main comparisons involving the ESS group; this is the case even with allowing for the impact of potential variable cluster sizes (cluster size variance of 49). A study of 600 would also likely provide around 50% and 80% power for the test of treatment by subgroup (CRSwNPs versus CRSsNPs) interaction for a target difference of 10 and 13.8 respectively after allowing for clustering in the surgical arm and equivalent variation in cluster sizes.

## Recruitment

### Participants in the internal pilot phase (MACRO Conversation Study)

During the internal recruitment pilot phase, potential trial participants will be invited to take part in an embedded qualitative study (MACRO Conversation study) designed to evaluate and optimise trial recruitment. All potential participants will receive a separate MACRO Conversation Study Participant Study Information Sheet to read whilst waiting in the outpatient department (prior to being seen in clinic).

### All participants (in both internal pilot phase and main trial phase)

Posters and flyers will be placed in the outpatient department, with information relating to the MACRO Trial. All potential trial participants will be seen in an outpatient clinic by the PI or Co-Investigator and RN/RP, where the aforementioned routine screening assessments will be carried out and the MACRO trial will be verbally introduced by the PI/Co-I and RN/RP. The participant will be informed that in addition to the routine screening assessments, a trial specific ECG scan (to exclude contraindications to clarithromycin) will need to be undertaken as well as a pregnancy test (urine test) if the potential participant is of childbearing potential; this will form part of the main trial consent form.

If interested in the trial, the patient will be provided with a copy of the MACRO PIS to take home and review*. The PIS will detail the exact nature of the study, what it will involve for the patient; the implications and constraints of the protocol and the known side effects and any risks involved in taking part. It will be clearly stated that the participant is free to withdraw from the study at any time for any reason without prejudice to future care, and with no obligation to give the reason for withdrawal. In such an event, the choice of treatment will be a matter for decision between patient and their clinical team.

The RN/RP should update the screening log and a MACRO sticker will be placed in the notes, to help identify the patient when returning to clinic. All patients will be allowed sufficient time (a minimum of 48 hours) to consider the trial and decide if they would like to take part. Screening logs must be kept up-to-date and fully completed at all times, and sent to the MACRO trial Office for review on a monthly basis.

*If the nasal endoscopy and SNOT-22 questionnaire confirm that the patient is a potential MACRO participant, but the patient has not received AMT as deemed by the PI/Co-I, the MACRO trial can still be introduced, and the participant can be given a copy of the PIS to take home. If, following AMT, symptom control has not been achieved and further treatment is deemed necessary, the patient will be seen in clinic once more and the MACRO trial discussed in depth.

## Assignment of interventions

### Sequence generation

Following consent, once trial eligibility has been confirmed, participants will be randomised into the trial by the PI, Co-I or RN/RP. Participants will be randomly allocated to the treatment options using an automated, web based secure randomisation system (RRAMP) provided by the Oxford Clinical Trials Research Unit (OCTRU) with 1:1:1 allocation ratio. The algorithm will stratify by the presence of polyps and centre using permuted blocks of varying size and the allocations will be generated by the trial statistician.

### Allocation concealment mechanism and implementation

Centrally managed randomisation will ensure allocation concealment and prevent selection bias. The participant’s identifiable information will be recorded on the randomisation form, and will be uploaded to an encrypted, separate database at the University of Oxford. For participants allocated to receive the placebo or antibiotic medication, they will be allocated a treatment pack number for the corresponding medication. Treatment pack numbers will be randomly generated to ensure allocation concealment and blinding.

### Blinding (masking)

Blinding of participants and medical staff will be maintained for the comparison of clarithromycin through an identical placebo, although unblinding of treatment allocation can be provided by the MACRO trial office in case of clinical need. Participants and medical staff will not be blinded to receiving surgery. At the end of each trial participant’s follow-up period of 6 months, the participant will return to normal NHS care. Those who remain symptomatic at this point will receive further treatment as defined by their ENT clinician, which may include being offered steroids, antibiotics or ESS, depending on which arm of the trial they were in. Patients who were allocated to either placebo or clarithromycin will not be told of their allocation at the end of their 6 month trial period, except in an emergency.

In the event of the need to treat a patient in an emergency following a SAR, the treating PI or medical doctor can break the code to see if the patient was on the active drug using the randomisation system. Under these exceptional circumstances the PI or member of the site team can unblind the participant using the CTU’s in-house RRAMP system. If unblinding is required for any other reasons apart from emergency, site staff must submit a request (through RRAMP) and the request will be reviewed, and if appropriate will be approved by a member of the central MACRO Trial Office. Where a serious adverse reaction (SAR) is not an emergency to treat but is deemed to be a suspected unexpected serious adverse reaction (SUSAR) then the CTU will break the code for reporting purposes without necessarily involving the PI. If on breaking the code it is found that the patient was actually on placebo the event will not be classed as a SUSAR..

## Data collection methods

### Confidentiality

All data will be handled in accordance with the General Data Protection Regulation (EU) 2016/679.

Participants joining MACRO will consent to giving identifiable information that will include their name, e-mail address and telephone contact numbers in order to allow completion of PROMs and resource use diaries electronically. If the participant does not wish to consent to electronic completion of documentation they will be asked instead to provide their home address and telephone number, so that the MACRO trial office can send the PROMs questionnaires and resource use diaries by post. This is coordinated centrally by the MACRO Trial Office. Participants’ identifiable information will be kept securely on a University of Oxford network database, which is separate to the clinical database.

Upon randomisation, data will be pseudonymised and a study number will be given to the participant. CRFs will not bear the participant’s name and the study number will be used for identification. Screening logs will list the participant’s initials, year of birth and trial ID to allow identification for site research staff when a participant returns to clinic.

### Data collection tools and source document identification

Clinical data will be collected from sites on trial specific paper CRFs, which will be completed by the local research teams and sent to the MACRO office for data entry. Data will be entered onto a validated installation of OpenClinica ([www.openclinica.com](http://www.openclinica.com)) which is held in a secure database and can only be accessed by authorised users via the OpenClinica application. The OpenClinica application resides on a webserver hosted and managed by Oxford University’s Medical Services Division IT Services department (<http://www.imsu.ox.ac.uk/>). Some CRF data may be entered electronically onto LimeSurvey. Participants will be requested to complete PROMs Questionnaires and resource utilisation diaries electronically using LimeSurvey on a weekly and three-monthly basis. A paper version will also be available for participants to complete, along with pre-paid envelopes. Text messaging and e-mail reminders to participants (who have consented to provide their telephone numbers and e-mail addresses) who have not returned/completed PROMs questionnaires and resource utilisation diaries will be used to maximise completeness of data. The MACRO Trial Office may also call participants if there are a number of outstanding PROMs questionnaires and resource utilisation diaries not completed by the participant.

If it becomes clear during the pilot phase that there is a low return rate for the prospective online weekly compliance diaries, the TMG will discuss whether to switch to also using retrospective data collection for this aspect of the trial, i.e. at the 6-week, 3-month and 6-month time points.

## statistical methods

### Statistical analysis

Full details of the Statistical Analysis Plan (SAP) will be agreed by the MACRO Programme Steering Committee (PSC) in advance of unblinding of the data. A summary of the planned analyses is presented here. Primary analysis will be according to randomised allocation irrespective of subsequent treatment compliance and conducted at 2-sided 5% significance level with 95% confidence intervals produced. No adjustment for multiple comparisons is planned as the comparisons relate to distinct clinical decisions (use or non-use of an antibiotic and surgery versus medical management of one form). Baseline data and participant flow information will be summarised without formal analysis. The primary outcome will be analysed using linear regression adjusted for baseline SNOT-22 and also the presence or not of polyps at baseline. Clustering by surgeon will also be accounted for where relevant using an appropriate method (e.g. cluster robust option in Stata). Secondary outcomes will be analysed similarly using generalised linear models.

#### Sensitivity and other planned analyses

The impact of missing primary outcome data will be assessed in sensitivity analyses e.g. multiple imputation approach as appropriate. Exploratory sub-group analyses will assess the impact of the presence of polyps under the treatment effect. Impact of compliance will be evaluated using a complier average causal effect (CACE) approach or similar.

### Interim analysis

No interim analyses are anticipated prior to completion of follow-up for the designated time points. This is due to the nature of the trial, including the primary outcome and the desire to have reasonable precision to assess the subgroups of CRSwNPs and CRSsNPs.

## Data monitoring

Details of the committee personnel can be found in appendix 1.

### Trial Management Group (TMG)

The TMG will include the Chief Investigator, Lead Collaborative Investigator, trial staff and members of the MACRO Programme. A member of the Sponsor team will also be invited to attend. The TMG will be responsible for overseeing the trial. Monthly TMG teleconferences will take place at the start of the trial, and at least two face-to-face meetings will take place annually.

The TMG will review recruitment figures, serious adverse events (SAEs) and substantial amendments to the protocol prior to submission to the REC and/or MHRA. All PIs will be kept informed of substantial amendments through their nominated responsible individuals. A TMG charter will be in place for the MACRO trial.

### Trial Steering Committee (TSC)

As MACRO forms part of a Programme of work, an overarching independent PSC is in place for the duration of the Programme. The PSC contains experienced members relevant to each specialist area of the MACRO programme and includes an experienced triallist, a clinician, a statistician, a qualitative researcher, a health economist, a GP and a patient. For the period of the MACRO trial, the PSC will assume the role of the TSC and will provide overall supervision of the trial. The TSC will review the recommendations of the (Independent) Data Safety Monitoring Committee (DSMC) and, on consideration of this information, recommend any appropriate amendments/actions for the trial as necessary. The TSC acts on behalf of the funder(s) and Sponsor.

### Data Safety Monitoring Committee (DSMC)

The role of the DSMC is to provide independent advice on data and safety aspects of the trial. Meetings of the Committee will be held annually to review safety data and any other issues that have arisen. A DSMC charter will be in place for the MACRO trial.

### Stopping rules

The trial may be stopped before completion for the following reasons:

- On the recommendation of the TSC or DSMC
- On the recommendation of the sponsor and CI

### Discontinuation/withdrawal of participants

#### Participant withdrawal from trial treatment

If a participant expresses their wish to withdraw from trial treatment, sites should explain the importance of remaining on trial follow-up (including completing the patient reported trial questionnaires and diaries) and seek permission to allow use of routine follow-up data to be used for trial purposes. The importance of safety follow-up should be emphasised to the participant in the PIS. The decision of the participant to withdraw from treatment must be recorded in the Change of Status CRF and medical notes. The participant may withhold their reason for withdrawal. However, if the participant gives a reason for their withdrawal from trial treatment, this should be recorded. If a participant allocated to either of the medical therapy arms requests to be given a non-trial treatment in addition to or instead of their trial treatment, this should also be recorded in the Change of Status CRF. If the participant wishes to withdraw from trial follow-up, a Discontinuation CRF should be completed. Where necessary for safety reasons e.g. if the participant requests to receive clarithromycin they will be unblinded at this point. Permission to continue to collect data will be sought irrespective of the treatment received.

#### Withdrawal of Consent to Data Collection

If a participant explicitly states that they do not wish to contribute further data to the trial, their decision must be respected and recorded in the Discontinuation CRF and medical notes. Any data already collected on the patient will be included for analysis.

#### Loss to follow-up

If a participant moves from the area, every effort should be made for the participant to be followed up at another participating trial site and for this new site to take over the responsibility for the participant. If a participant is lost to follow-up at a site, every effort should be made to contact the participant’s GP to obtain information on the participant’s status.

### Replacements

There will not be any replacement of individuals due to non-compliance with the treatment allocation or the study protocol more generally (e.g. attending a clinical visit or completing a questionnaire) as the trial is using an intention-to-treat analysis. It should be noted that a randomisation error (e.g. incorrectly randomising an individual a second time) will not be considered a valid randomisation.

### Definition of End of Trial

The expected duration of recruitment and follow-up for the primary outcome data collection point is 52 months from recruitment of the first patient. For regulatory purposes the trial will be deemed ended on the date when all data has been received, cleaned and queries have been resolved at each site. Subject to additional funding, trial follow-up will continue for a further 40-60 months after the initial 52 months’ trial duration, to enable completion of long term follow-up for all participants who have given their consent (up to 5 years). For regulatory purposes the trial will then be deemed ended on the date when all long term follow-up data has been received and cleaned.

## Harms

### Recording of Adverse Events (AEs) and Serious Adverse Events (SAEs)

#### Events in relation to IMP

The most frequent and common adverse reactions related to clarithromycin therapy for adults are:

- Abdominal pain
- Diarrhoea
- Nausea
- Vomiting
- Dysgeusia
- Headache
- Insomnia
- Dyspepsia
- Rash
- Hyperhidrosis
- Liver function test abnormal

These adverse reactions are usually mild in intensity and are consistent with the known safety profile of macrolide antibiotics. Adverse events will be recorded in the hospital notes in the first instance. All adverse events will be recorded with clinical symptoms and accompanied with a simple brief description of the event, including dates as appropriate. If any of the listed events above occur within its frequency and severity, these should also be detailed in the 3 or 6 month clinic visit Follow-up CRF when the patient is seen by the PI/Co-I or RN/RP. If the events are deemed to be more severe than expected the site must complete both an AE CRF and if the event satisfies the definition of a Serious Adverse Reaction (SAR), the site must also complete an SAE form.

For all other events that are related to the IMP/placebo, the site must complete an AE CRF. If the event satisfies the definition of a Serious Adverse Reaction (SAR), the site must also complete an SAE form.

#### Events in relation to ESS

The following adverse events are possible following ESS:

- Post-op bleeding needing nasal packing or readmission (1 in 200)
- Post op infection requiring antibiotics (1 in 15)
- Bruising around eye (1 in 500)
- Cerebrospinal fluid (CSF) leak (damage to the skull base which may lead to meningitis), requiring repair, either at time of surgery, causing delayed discharge or later, causing further admission (1 in 1500)
- Major orbital injury leading to double vision or blindness (<1 in 10,000)

These events, and all other events in relation to the ESS will be recorded on the Post-procedure CRF. If the event satisfies the definition of a SAE, the site must also complete an SAE form. A delayed discharge will not be considered an SAE if the participant’s hospital stay is less than 24 hours.

### Reporting of Adverse Events and Serious Adverse Events

The delivery of the Sponsor’s responsibility for Pharmacovigilance has been delegated to the Surgical Interventions Trials Unit (SITU), within the Oxford Clinical Trials Research Unit (OCTRU), Oxford University.

#### Adverse Events in relation to the IMP

Any adverse event or reaction (be it expected, more severe than expected, or unexpected) should be recorded from the Baseline Assessment.

- For those adverse events or reactions that are deemed most frequent and common, no AE CRF is required. When the participant is seen in clinic at their 3 and 6 month post-randomisation follow-up visit, these common adverse reactions will be recorded by the PI/Co-I or RN/RP in the follow-up CRF.
- If the adverse event or reaction is more severe than expected, an AE CRF must be completed by the PI/Co-I or RN/RP
- If the adverse event or reaction satisfies the definition of a Serious Adverse Reaction (SAR), an SAE form must also be completed by the PI/Co-I or RN/RP

Any AE (that is more severe than expected) or SAE that is considered related to the trial medication or surgery intervention as judged by a medically qualified investigator will be followed either until resolution, or the event is considered stable.

#### Serious Adverse Events in relation to the IMP

The PI/Co-I or RN/RP should complete a paper SAE form. The following information will be recorded: description, date of onset and end date, severity, assessment of causality by trial medication, other suspect drug or device and action taken. Follow-up information should be provided as necessary. Hospitalisation as part of the standard clinical care and trial visits will not be reported as an SAE, unless extended beyond expected lengths.

#### Adverse Events in relation to ESS

- AE CRFs do not need to be completed for adverse events in relation to ESS, however they should be recorded from the Baseline Assessment and detailed in the 3 and 6 month post-randomisation follow-up visit CRF by the PI/Co-I or RN/RP.
- If the adverse event satisfies the definition of a Serious Adverse Event, the PI/Co-I or RN/RP must complete an SAE form. If the SAE that is considered related to the trial medication or surgery intervention as judged by a medically qualified investigator will be followed either until resolution, or the event is considered stable.

#### Serious Adverse Events in relation to ESS

The PI/Co-I or RN/RP should complete a paper SAE form. The following information will be recorded: description, date of onset and end date, severity, assessment of causality by trial medication, other suspect drug or device and action taken. Follow-up information should be provided as necessary. Hospitalisation as part of the standard clinical care and trial visits will not be reported as an SAE, unless extended beyond expected lengths. CSF leak leading to meningitis (and hospitalisation) or major orbital injury leading to double vision or blindness must be recorded as SAEs. A delayed discharge for participants undergoing ESS will not be considered an SAE if the participant’s hospital stay is less than 24 hours.

The severity of events (both in relation to the IMP or ESS) will be assessed on the following scale: 1 = mild, 2 = moderate, 3 = severe:

| **Category** | **Definition** |
| --- | --- |
| Mild | The adverse event does not interfere with the volunteer’s daily routine, and does not require intervention; it causes slight discomfort |
| Moderate | The adverse event interferes with some aspects of the volunteer’s routine, or requires intervention, but is not damaging to health; it causes moderate discomfort |
| Severe | The adverse event results in alteration, discomfort or disability which is clearly damaging to health |

The SAE form must be sent via secure e-mail to [situ.oxford@nhs.net](mailto:situ.oxford@nhs.net) within 24 hours of the local trial team becoming aware of the event.

## Auditing

### Processing of SAE forms and assessment of expectedness

The Trial Office will process SAEs as per instructions in the relevant safety SOP by OCTRU. SAEs will be reviewed by the Nominated Person (NP) and any queries clarified with the site. The NP will also perform the central assessment of expectedness. For SAEs related to the IMP/placebo, expectedness will be assessed according to the current approved summary of product characteristics for clarithromycin.

#### SUSAR Reporting

All Suspected Unexpected Serious Adverse Reactions (SUSARs) will be reported by SITU to the relevant Competent Authority (MHRA) and to the REC and other parties as applicable. For fatal and life-threatening SUSARs, this will be done no later than 7 calendar days after the Sponsor or delegate is first aware of the reaction. Any additional relevant information will be reported within 8 calendar days of the initial report. All other SUSARs will be reported within 15 calendar days. Principal Investigators will be informed of all SUSARs for the relevant IMP for all studies using the same IMP with the same Sponsor, whether or not the event occurred in the current trial. Line listings will be sent to sites every 3 months and any new SUSARs discussed at each TMG meeting.

#### Development Safety Update Reports (DSUR)

SITU, on behalf of the CI and Sponsor will submit (in addition to the expedited reporting above) Development Safety Update Reports (DSURs) once a year throughout the clinical trial, or on request, to the Competent Authority (MHRA in the UK), Ethics Committee, Health Research Authority (HRA) (where required) and Sponsor. The report will be submitted within 60 days of the Developmental International Birth Date (DIBD) of the trial each year until the trial is declared ended.

#### Notification of Deaths

In the unlikely event of a death, the site must complete a Notification of Death Form and send it to the Trial Office within 24 hours of the site becoming aware. If the death is the outcome of a SAE it must be reported both as a SAE (within 24 hrs) and a Notification of Death Form also completed.

#### Pregnancy

Female participants of childbearing potential will be counselled appropriately by the PI/Co-I during the clinic visit and requested to use a reliable method of contraception (e.g. hormonal contraceptive patch, intrauterine device, physical barrier or abstinence if preferred and usual lifestyle of the patient) for the duration of the trial.

##### IMP/Placebo Arm

If a female participant becomes pregnant during the 3 month active IMP/placebo period, a completed trial specific Pregnancy Reporting Form should be sent to the secure e-mail address: [situ.oxford@nhs.net](mailto:situ.oxford@nhs.net) within 24 hours of the site becoming aware of the event. The local Principal Investigator will respond to any queries raised by the Trial Office as soon as possible. The participant should be unblinded and asked to stop taking the medication, but will be requested to remain in the study, and followed-up as per protocol. The site should complete a Change of Status CRF and send it to the MACRO Trial Office.

In the event that a female participant becomes pregnant outside of the 3-month active/placebo period, the site must only complete a Pregnancy Notification Form and submit it to the Trial Office. No further action is required by the site.

##### ESS Arm

If the female participant becomes pregnant **before** receiving ESS, the participant should be **withdrawn from trial treatment**, but be requested to **remain in the trial and undergo trial follow-up as per protocol**. The site should complete a ‘Discontinuation CRF’ and ‘Change of Status CRF’ and send these in to the MACRO Trial Office. In the event that a female participant becomes pregnant **after** receiving ESS, the site must only complete a Pregnancy Notification Form and submit it to the Trial Office. No further action is required by the site and the participant will undergo trial follow-up as per protocol.

#### Overdose

Sites will record details of reported overdoses of IMP/Placebo on the deviation log and inform the trial manager at Oxford CTU as soon as possible after being made aware of the information. An overdose is defined as 3 or more tablets in 24 hours for 1 day or more of the IMP/Placebo. If an overdose has taken place, the patient will suspend medication for at least 48 hours, and resume the previous dose as soon as possible afterwards, at the discretion of the PI. If an AE occurs, and it is deemed to be more severe than expected (see section 9.2) the site must complete both an AE CRF and if the event satisfies the definition of a Serious Adverse Reaction (SAR), the site must also complete an SAE form. The patient will continue in trial if no SAE has occurred. In the event that an SAE is associated with the overdose the SAE reporting procedure in addition should be followed. Details of the overdose will documented in the SAE form. The trials office will notify the sponsor that an overdose associated with a SAE has occurred.

# Ethics and dissemination

## Research ethics approval

Please see below.

## Protocol amendments

The sponsor will ensure that the trial protocol, participant information sheet, consent form, GP letter and submitted supporting documents have been approved by the appropriate regulatory body (MHRA in UK) and an appropriate research ethics committee, prior to any participant recruitment. The protocol, all other supporting documents including and agreed amendments, will be documented and submitted for ethical and regulatory approval as required. Amendments will not be implemented prior to receipt of the required approval(s).

## Consent or assent

Informed consent will be obtained from all participants. Each potential participant will be telephoned by the RN/RP **at least** 48 hours after being given the MACRO PIS. The RN/RP can answer any initial questions about the trial over the phone, and also invite the patient to return to clinic to discuss taking part in the MACRO trial in more depth. It must be recorded in the medical notes when the participant information sheet (PIS) has been given to the participant as well as what version it is.

**The follow-up outpatient clinic appointment can either by conducted by the PI or Co-I, or the RN/RP**. As well as discussing details of the MACRO trial, the following elements of the consent process should be outlined:-

1. A trial specific ECG must be carried out to confirm eligibility and consent is required for this
2. A pregnancy test must be undertaken by females of childbearing potential and consent is required for this
3. The patient will be requested to consent to provide their e-mail address and mobile phone number to enable electronic completion of PROMs questionnaires and resource utilisation diaries. If the patient does not wish to consent to electronic completion of documentation, they will be asked instead to provide their home address and telephone number, so that the MACRO trial office can send the PROMs questionnaires and resource utilisation diaries by post.
4. The participant has the option to consent to being contacted in the future about the MACRO Trial, or participating in other research studies (e.g. by giving tissue/blood/mucus samples)
5. The participant has the option to consent to an interview with the Qualitative Researcher as part of the mixed methods evaluation
6. The participant has the option to consent to being followed-up annually for a period of up to 5 years, as part of the larger MACRO Programme of work. Participants participating in long-term follow-up will be requested to complete the SNOT-22 and EQ-5D-5L validated questionnaires, and a short questionnaire on-line (a paper version is also available)

A trained RN/RP, the local PI, Co-I or another appropriately qualified member of the research team, will obtain informed consent. The person taking informed consent will be GCP trained, suitably qualified and experienced, and will have been delegated this duty by the CI/ PI on the Staff Signature and Delegation Log. The Investigator or designee will explain that patients are under no obligation to enter the trial and that they can withdraw at any time during the trial without providing a reason. No clinical trial procedures will be conducted prior to the participant giving consent by signing the ICF. Consent will not denote enrolment into the trial.

A copy of the signed ICF will be given to the participant. The original signed form will be retained in the trial file at site and a copy placed in the medical notes. The PIS and ICF be reviewed and updated if necessary throughout the trial (e.g. where new safety information becomes available) and participants will be re-consented as appropriate.

## Confidentiality

See above section on data management.

## Declaration of interests

There are no financial or other competing interests for the research team.

## Access to data

The research team (see appendix 1) will have access to the final trial database.

## Ancillary and post-trial care

At the completion of the trial the patient will remain under normal clinical care provided by the primary care physician and treating ENT surgeon.

## Dissemination policy

As part of the wider body of work of the MACRO programme, we anticipate dissemination through traditional channels including conference presentations, abstracts and open-access peer reviewed publications. These will be targeted at both specialist and generalist groups, facilitated by roles held by members of our collaboration in relevant professional societies and guideline producing bodies. However, beyond informal and specialist publications, we also plan to use wider communication channels to allow larger scale dissemination to the general public. When our PPI panel has facilitated a patient-friendly and comprehensible summary of study findings, these will be provided to all study participants by mailings and to the wider public via a study website. We will maximize non-professional dissemination through a number of multimedia outlets, including targeted mailshots, press releases to medical and general journalists, health information websites and communications media, evidence-based interest groups, conference presentations, social media and patient interest groups/medical charities such as Fifth Sense and Asthma UK. We anticipate the results will lead to the production of new guidelines which will be made available through websites such as ENT UK and the Royal College of Surgeons.

# Discussion

The MACRO trial is part of the MACRO Programme funded by the National Institute of Health Research Programme Grants for Applied Research funding stream. The overarching Programme is run by a Programme Management Group. As listed below in appendix 1, the trial is managed by the Trial Management Group and has independent oversight from the Programme Steering Committee and the Data Safety and Monitoring Committee. Day to day trial administration is the responsibility of the Trial Manager in liaison with the Chief Investigators. The Trial Manager is supported by colleagues at the Oxford Surgical Intervention Trials Unit, which is part of the UKCRC Clinical Trials Unit, OCTRU – see appendix 1. The sites for the trial were selected based on volume of cases and prior engagement of the PIs in the research group at the British Rhinological Society, but selecting a mix of teaching hospital and district general hospital environments over a wide geographical location.

The effectiveness of antibiotics will be established through the trial, reducing unnecessary usage and potential morbidity. With respect to ESS, if it is shown to be clinically and cost effective, the trial may encourage earlier intervention. In contrast, if shown to be ineffective, this should achieve significant reductions in surgery rates. The trial results will feed into the other components of the MACRO research programme to establish best practice for the management of adults with Chronic Rhinosinusitis (CRS) and design the ideal patient pathway across primary and secondary care.

# Trial status

The trial is currently in setup with recruitment expected to begin by December 2018.

# Declarations

## Ethics approval and consent to participate:

Version 3.0 of the protocol (dated 31^st^ August 2018) was approved centrally by the North East - Newcastle & North Tyneside 2 Research Ethics Committee on 20th September 2018. The Medicines and Healthcare products Regulatory Agency gave approval for a clinical trial authorisation on 13^th^ July 2018. The trial manager has informed the Health Regulation Authority for each of the sites to be included in the trial. Local site Research & Development departments will confirm capacity and capability and the Oxford SITU will issue a green light to proceed before recruitment at each site can commence. Informed consent will be obtained from all study participants as detailed above.

## Consent for publication

Not applicable

## Availability of data and material

Not applicable

## Competing interests

None declared

## Funding

See page 45

## Authors' contributions

All authors:

CP, SlC, DB, JC, WS, SM, CC, MT, PL, JV, VL, HB, AS, SD, SDe, JCa, JB, CH

contributed to the trial design and final manuscript through and membership of the MACRO Programme Management Group or the Trial Management Group.

## Acknowledgements

Edmund Wyatt and Surjeet Singh, Oxford SITU

Wider members of the MACRO Programme Team

NeilMed for provision of the sinus irrigation bottles for the trial

Keith Boland as sponsor representative for UCL

Jane Woods and Teresa Ferreira for research nurse feedback on trial set up

## Trial status:

Protocol Version V3.0 dated 31st August 2018; Sponsor protocol number: 14/0644

The trial is due to commence recruitment in November 2018 and is due to complete in 2022.

# References

1. Hastan D, Fokkens WJ, Bachert C, Newson RB, Bislimovska J, Bockelbrink A, Bousquet PJ, Brozek G, Bruno A, Dahlen SE *et al*: **Chronic rhinosinusitis in Europe--an underestimated disease. A GA(2)LEN study**. *Allergy* 2011, **66**(9):1216-1223.

2. Gliklich RE, Metson R: **The health impact of chronic sinusitis in patients seeking otolaryngologic care**. *Otolaryngol Head Neck Surg* 1995, **113**(1):104-109.

3. Gulliford MC, Dregan A, Moore MV, Ashworth M, Staa T, McCann G, Charlton J, Yardley L, Little P, McDermott L: **Continued high rates of antibiotic prescribing to adults with respiratory tract infection: survey of 568 UK general practices**. *BMJ Open* 2014, **4**(10):e006245.

4. **Hospital Episode Statistics**. In*.*: Department of Health; 2013.

5. Lange B, Holst R, Thilsing T, Baelum J, Kjeldsen A: **Quality of life and associated factors in persons with chronic rhinosinusitis in the general population: A prospective questionnaire and clinical cross-sectional study**. *Clin Otolaryngol* 2013, **38**(6):474-480.

6. Sahlstrand-Johnson P, Ohlsson B, Von Buchwald C, Jannert M, Ahlner-Elmqvist M: **A multi-centre study on quality of life and absenteeism in patients with CRS referred for endoscopic surgery**. *Rhinology* 2011, **49**(4):420-428.

7. Erskine SE, Hopkins C, Clark A, Anari S, Robertson A, Sunkaraneni S, Wilson JA, Beezhold J, Philpott CM: **Chronic rhinosinusitis and mood disturbance**. *Rhinology* 2017, **55**(2):113-119.

8. Goetzel RZ, Hawkins K, Ozminkowski RJ, Wang S: **The health and productivity cost burden of the "top 10" physical and mental health conditions affecting six large U.S. employers in 1999**. *J Occup Environ Med* 2003, **45**(1):5-14.

9. Fokkens WJ, Lund VJ, Mullol J, Bachert C, Alobid I, Baroody F, Cohen N, Cervin A, Douglas R, Gevaert P *et al*: **European Position Paper on Rhinosinusitis and Nasal Polyps 2012**. *Rhinol Suppl* 2012(23):3 p preceding table of contents, 1-298.

10. Vennik J, Eyles C, Thomas DM, Hopkins C, Little P, Blackshaw H, Schilder AG, Boardman J, Philpott CM: **Management strategies for chronic rhinosinusitis: A qualitative study of GP and ENT specialist views of current practice in the UK**. *BMJ Open* 2018, **In press**.

11. Hopkins C, McCombe A, Philpott C, Hern J, Blythe J, Thomas D, Bohm N, Bhalla R, Lund V, Veldtman D *et al*: **Commissioning Guide: Rhinosinusitis**. In*.*: ENT UK/Royal College of Surgeons of England; 2013.

12. Akkerman AE, Kuyvenhoven MM, van der Wouden JC, Verheij TJ: **Prescribing antibiotics for respiratory tract infections by GPs: management and prescriber characteristics**. *Br J Gen Pract* 2005, **55**(511):114-118.

13. Cervin A, Wallwork B: **Macrolide therapy of chronic rhinosinusitis**. *Rhinology* 2007, **45**(4):259-267.

14. Wallwork B, Coman W, Mackay-Sim A, Greiff L, Cervin A: **A double-blind, randomized, placebo-controlled trial of macrolide in the treatment of chronic rhinosinusitis**. *Laryngoscope* 2006, **116**(2):189-193.

15. Videler WJ, Badia L, Harvey RJ, Gane S, Georgalas C, van der Meulen FW, Menger DJ, Lehtonen MT, Toppila-Salmi SK, Vento SI *et al*: **Lack of efficacy of long-term, low-dose azithromycin in chronic rhinosinusitis: a randomized controlled trial**. *Allergy* 2011, **66**(11):1457-1468.

16. Pynnonen MA, Venkatraman G, Davis GE: **Macrolide therapy for chronic rhinosinusitis: a meta-analysis**. *Otolaryngol Head Neck Surg* 2013, **148**(3):366-373.

17. Cervin A, Wallwork B: **Efficacy and safety of long-term antibiotics (macrolides) for the treatment of chronic rhinosinusitis**. *Current allergy and asthma reports* 2014, **14**(3):416.

18. Piromchai P, Thanaviratananich S, Laopaiboon M: **Systemic antibiotics for chronic rhinosinusitis without nasal polyps in adults**. *Cochrane Database Syst Rev* 2011(5):CD008233.

19. Baguley C, Brownlow A, Yeung K, Pratt E, Sacks R, Harvey R: **The fate of chronic rhinosinusitis sufferers after maximal medical therapy**. *International forum of allergy & rhinology* 2014, **4**(7):525-532.

20. Young LC, Stow NW, Zhou L, Douglas RG: **Efficacy of medical therapy in treatment of chronic rhinosinusitis**. *Allergy Rhinol (Providence)* 2012, **3**(1):e8-e12.

21. Hopkins C, Andrews P, Holy CE: **Does time to endoscopic sinus surgery impact outcomes in chronic rhinosinusitis? Retrospective analysis using the UK clinical practice research data**. *Rhinology* 2015, **53**(1):18-24.

22. Hopkins C, Rimmer J, Lund V: **Does time to endoscopic sinus surgery impact outcomes in Chronic Rhinosinusitis? Prospective findings from the National Comparative Audit of Surgery for Nasal Polyposis and Chronic Rhinosinusitis**. *Rhinology* 2014, **52**.

23. NICE: **Database of Uncertainties of Treatments**. In*.*

24. Orlandi RR, Kingdom TT, Hwang PH, Smith TL, Alt JA, Baroody FM, Batra PS, Bernal-Sprekelsen M, Bhattacharyya N, Chandra RK *et al*: **International Consensus Statement on Allergy and Rhinology: Rhinosinusitis**. *International forum of allergy & rhinology* 2016, **6 Suppl 1**:S22-209.

25. Rudmik L, Soler ZM, Hopkins C, Schlosser RJ, Peters A, White AA, Orlandi RR, Fokkens WJ, Douglas R, Smith TL: **Defining appropriateness criteria for endoscopic sinus surgery during management of uncomplicated adult chronic rhinosinusitis: a RAND/UCLA appropriateness study**. *International forum of allergy & rhinology* 2016, **6**(6):557-567.

26. Head K, Chong LY, Piromchai P, Hopkins C, Philpott C, Schilder AG, Burton MJ: **Systemic and topical antibiotics for chronic rhinosinusitis**. *Cochrane Database Syst Rev* 2016, **4**:CD011994.

27. Head K, Chong LY, Hopkins C, Philpott C, Burton MJ, Schilder AG: **Short-course oral steroids alone for chronic rhinosinusitis**. *Cochrane Database Syst Rev* 2016, **4**:CD011991.

28. Head K, Chong LY, Hopkins C, Philpott C, Schilder AG, Burton MJ: **Short-course oral steroids as an adjunct therapy for chronic rhinosinusitis**. *Cochrane Database Syst Rev* 2016, **4**:CD011992.

29. Chong LY, Head K, Hopkins C, Philpott C, Burton MJ, Schilder AG: **Different types of intranasal steroids for chronic rhinosinusitis**. *Cochrane Database Syst Rev* 2016, **4**:CD011993.

30. Chong LY, Head K, Hopkins C, Philpott C, Schilder AG, Burton MJ: **Intranasal steroids versus placebo or no intervention for chronic rhinosinusitis**. *Cochrane Database Syst Rev* 2016, **4**:CD011996.

31. Chong LY, Head K, Hopkins C, Philpott C, Glew S, Scadding G, Burton MJ, Schilder AG: **Saline irrigation for chronic rhinosinusitis**. *Cochrane Database Syst Rev* 2016, **4**:CD011995.

32. Dalziel K, Stein K, Round A, Garside R, Royle P: **Systematic review of endoscopic sinus surgery for nasal polyps**. *Health technology assessment* 2003, **7**(17):iii, 1-159.

33. Dalziel K, Stein K, Round A, Garside R, Royle P: **Endoscopic sinus surgery for the excision of nasal polyps: A systematic review of safety and effectiveness**. *American journal of rhinology* 2006, **20**(5):506-519.

34. Rimmer J, Fokkens W, Chong LY, Hopkins C: **Surgical versus medical interventions for chronic rhinosinusitis with nasal polyps**. *Cochrane Database Syst Rev* 2014, **12**:CD006991.

35. Sharma R, Lakhani R, Rimmer J, Hopkins C: **Surgical interventions for chronic rhinosinusitis with nasal polyps**. *Cochrane Database Syst Rev* 2014, **11**:CD006990.

36. **Duration of Antibiotic Therapy as Part of Maximal Medical Therapy for Chronic Rhinosinusitis** [<https://clinicaltrials.gov/ct2/show/NCT01825408>]

37. **Azithromycin for Patients With Chronic Rhinosinusitis Failing Medical and Surgical Therapy (AZI-CRS)** [<https://clinicaltrials.gov/ct2/show/NCT02307825>]

38. Vennik J, Eyles C, Thomas DM, Hopkins C, Little P, Blackshaw H, Schilder AG, Savage I, Philpott CM: **Chronic rhinosinusitis: a qualitative study of patient views and experiences of current management in primary and secondary care.** *BMJ Open* 2018, **Under review**.

39. Lourijsen ES, de Borgie CA, Vleming M, Fokkens WJ: **Endoscopic sinus surgery in adult patients with chronic rhinosinusitis with nasal polyps (PolypESS): study protocol for a randomised controlled trial**. *Trials* 2017, **18**(1):39.

40. Anand VK: **Epidemiology and economic impact of rhinosinusitis**. *Ann Otol Rhinol Laryngol Suppl* 2004, **193**:3-5.

41. Bhattacharyya N: **The economic burden and symptom manifestations of chronic rhinosinusitis**. *American journal of rhinology* 2003, **17**(1):27-32.

42. Lodato EM, Kaplan W: **Priority Medicines for Europe and the World "A Public Health Approach to Innovation": Background Paper 6.1 Antimicrobial resistance**. In*.*: World Health Organisation; 2013.

43. Davies SC, Gibbens N: **UK Five Year Antimicrobial Resistance Strategy 2013 to 2018**. In*.*: Department of Health and Department for Environment, Food & Rural Affairs; 2013.

44. Gibbins NE, Theokli C, Hopkins C: **Time to reconsider guidelines on clarithromycin in chronic rhinosinusitis?** *Bmj* 2013, **346**:f2678.

45. Soni-Jaiswal A, Philpott C, Hopkins C: **The impact of commissioning for rhinosinusitis in England**. *Clin Otolaryngol* 2015, **40**(6):639-645.

46. Philpott C, Hopkins C, Erskine S, Kumar N, Robertson A, Farboud A, Ahmed S, Anari S, Cathcart R, Khalil H *et al*: **The burden of revision sinonasal surgery in the UK-data from the Chronic Rhinosinusitis Epidemiology Study (CRES): a cross-sectional study**. *BMJ Open* 2015, **5**(4):e006680.

47. Hopkins C, Slack R, Lund V, Brown P, Copley L, Browne J: **Long-term outcomes from the English national comparative audit of surgery for nasal polyposis and chronic rhinosinusitis**. *Laryngoscope* 2009, **119**(12):2459-2465.

48. Soni-Jaiswal A, Philpott CM, Hopkins C: **The Impact of Commissioning for Rhinosinusitis in England**. *Clinical Otolaryngology* 2015, **Under review**.

49. Hopkins C, Browne JP, Slack R, Lund V, Topham J, Reeves B, Copley L, Brown P, van der Meulen J: **The national comparative audit of surgery for nasal polyposis and chronic rhinosinusitis**. *Clin Otolaryngol* 2006, **31**(5):390-398.

50. Soler ZM, Smith TL: **Quality-of-life outcomes after endoscopic sinus surgery: how long is long enough?** *Otolaryngol Head Neck Surg* 2010, **143**(5):621-625.

51. Davidson R, Peloquin L: **Anti-inflammatory effects of the macrolides**. *J Otolaryngol* 2002, **31 Suppl 1**:S38-40.

52. Zalewska-Kaszubska J, Gorska D: **Anti-inflammatory capabilities of macrolides**. *Pharmacol Res* 2001, **44**(6):451-454.

53. Kanoh S, Rubin BK: **Mechanisms of action and clinical application of macrolides as immunomodulatory medications**. *Clin Microbiol Rev* 2010, **23**(3):590-615.

54. Fraschini F, Scaglione F, Pintucci G, Maccarinelli G, Dugnani S, Demartini G: **The diffusion of clarithromycin and roxithromycin into nasal mucosa, tonsil and lung in humans**. *J Antimicrob Chemother* 1991, **27 Suppl A**:61-65.

55. Zimmermann P, Ziesenitz VC, Curtis N, Ritz N: **The Immunomodulatory Effects of Macrolides-A Systematic Review of the Underlying Mechanisms**. *Front Immunol* 2018, **9**:302.

56. Genoway KA, Philpott CM, Javer AR: **Pathogen yield and antimicrobial resistance patterns of chronic rhinosinusitis patients presenting to a tertiary rhinology centre**. *J Otolaryngol Head Neck Surg* 2011, **40**(3):232-237.

57. Ikawa K, Kikuchi E, Kikuchi J, Nishimura M, Derendorf H, Morikawa N: **Pharmacokinetic modelling of serum and bronchial concentrations for clarithromycin and telithromycin, and site-specific pharmacodynamic simulation for their dosages**. *J Clin Pharm Ther* 2014, **39**(4):411-417.

58. Hopkins C, Philpott C, Carrie S, Blythe J, Thomas DM, Little P, Scadding G, Wilkes S, Swift A, Saleh H: **Commissioning Guide: Rhinosinusitis.** . In*.* London: ENT UK/Royal College of Surgeons of England; 2016.

59. Ragab SM, Lund VJ, Scadding G: **Evaluation of the medical and surgical treatment of chronic rhinosinusitis: a prospective, randomised, controlled trial**. *Laryngoscope* 2004, **114**(5):923-930.

60. Hashiba M, Baba S: **Efficacy of long-term administration of clarithromycin in the treatment of intractable chronic sinusitis**. *Acta Otolaryngol Suppl* 1996, **525**:73-78.

61. Lasso A, Masoudian P, Quinn JG, Cowan J, Labajian V, Bonaparte JP, Kilty S: **Long-term low-dose macrolides for chronic rhinosinusitis in adults - a systematic review of the literature**. *Clin Otolaryngol* 2017, **42**(3):637-650.

62. Wong AYS, Chan EW, Anand S, Worsley AJ, Wong ICK: **Managing Cardiovascular Risk of Macrolides: Systematic Review and Meta-Analysis**. *Drug Saf* 2017, **40**(8):663-677.

63. Cheng YJ, Nie XY, Chen XM, Lin XX, Tang K, Zeng WT, Mei WY, Liu LJ, Long M, Yao FJ *et al*: **The Role of Macrolide Antibiotics in Increasing Cardiovascular Risk**. *J Am Coll Cardiol* 2015, **66**(20):2173-2184.

64. Bewick J, Ahmed S, Carrie S, Hopkins C, Sama A, Sunkaraneni V, Woods J, Morris S, Erskine S, Philpott CM: **The value of a feasibility study into long-term macrolide therapy in chronic rhinosinusitis**. *Clin Otolaryngol* 2017, **42**(1):131-138.

65. Luo Q, Chen F, Liu W, Li Z, Xu R, Fan Y, Chen R, Xu Y, Liu Z, Xu G *et al*: **Evaluation of long-term clarithromycin treatment in adult Chinese Patients with chronic rhinosinusitis without nasal polyps**. *ORL J Otorhinolaryngol Relat Spec* 2011, **73**(4):206-211.

66. Hopkins C, Gillett S, Slack R, Lund VJ, Browne JP: **Psychometric validity of the 22-item Sinonasal Outcome Test**. *Clin Otolaryngol* 2009, **34**(5):447-454.

# Appendix 1: Trial registration, Protocol Version, Funding details, Roles and Responsibilities

## Trial registration

EudraCT no: 2018-001100-11 pending confirmation from EudraCT

Sponsor protocol number: 14/0644

## Protocol version

V3.0 dated 31st August 2018

## Funding

National Institute of Health Research (NIHR): Programme Grant for Applied Research Stream (PGfAR) - Reference Number: RP-PG-0614-20011

## Roles and responsibilities

Chief investigator: Professor Carl Philpott

- Norwich Medical School, University of East Anglia, Norwich, UK
- James Paget University Hospital NHS Foundation Trust, Gorleston, UK

Lead Collaborative Investigator: Professor Claire Hopkins

- Guy's and St Thomas' NHS Foundation Trust, London, UK

Sponsor Representative: Keith Boland

- Joint Research Office, UCL, London, UK

The sponsor was involved in reviewing and approving the version of the protocol submitted to the research ethics committee. The funder had no role in the writing of the protocol. The decision to submit the protocol for publication was taken by the MACRO Programme Joint Chief Investigators in conjunction with the Programme Manager and with the consensus of the protocol authors.

## Composition, roles, and responsibilities of relevant trial management groups

| Name | Role | Affiliation | Contact details |
| --- | --- | --- | --- |
| Core Trial Management Team | | | |
| Steffi le Conte | Clinical Trial Manager | SITU, University of Oxford | steffi.leconte@nds.ox.ac.uk |
| Ed Wyatt | Clinical Trial Data Coordinator | SITU, University of Oxford | edmund.wyatt@nds.ox.ac.uk |
| Jonathan Cook | Co-Applicant, Associate Professor, Centre for Statistics in Medicine (CSM) | CSM, University of Oxford | jonathan.cook@ndorms.ox.ac.uk |
| William Sones | Trial Statistician | CSM, University of Oxford | william.sones@ndorms.ox.ac.uk |
| Damian Haywood | Operational Lead | SITU, University of Oxford | damian.haywood@nds.ox.ac.uk |
| Wider Trial Management Team | | | |
| Helen Blackshaw | Co-Applicant, MACRO Programme Manager | evidENT, UCL | h.blackshaw@ucl.ac.uk |
| Michelle Tu | MACRO Programme Manager (maternity cover) | evidENT, UCL | m.tu@ucl.ac.uk |
| Anne Schilder | Co-Applicant, Professor of Otolaryngology | evidENT, UCL | a.schilder@ucl.ac.uk |
| Valerie Lund | Co-Applicant, Professor of Rhinology | UCL | v.lund@ucl.ac.uk |
| Kim Airey | MACRO Programme Administrator | evidENT, UCL | k.airey@ucl.ac.uk |
| Stephen Morris | Co-Applicant, Lead Health Economist | UCL | steve.morris@ucl.ac.uk |
| Caroline Clarke | Health Economist | UCL | caroline.clarke@ucl.ac.uk |
| Mike Thomas | Co-Applicant, Primary Care Lead | University of Southampton | D.M.Thomas@soton.ac.uk |
| Jane Vennik | Lead Qualitative Researcher | University of Southampton | J.Vennik@soton.ac.uk |
| David Beard | Co-Applicant, Co-Director, Surgical Intervention Trials Unit (SITU) | SITU, University of Oxford | david.beard@ndorms.ox.ac.uk |
| Jim Boardman | Co-Applicant, Patient Contributor | Fifth Sense | jim@boardmanfamily.freeserve.co.uk |
| Patrick Julier | Lead Programmer | OCTRU, University of Oxford | patrick.julier@ndorms.ox.ac.uk |
| IMP manufacturer | GSTT Pharmaceuticals | Guy's Hospital, 13th Floor,  Great Maze Pond, London, SE1 9RT | PMUclinicaltrials@gstt.nhs.uk |

| **Programme (Trial) Steering Committee** | | |
| --- | --- | --- |
| **Jane Blazeby** | PSC Independent Chair | Director of Bristol Surgical Trials Centre, University of Bristol |
| **Janet Wilson** | PSC Independent Member | Professor of Otolaryngology, Newcastle University |
| **Christie Cabral** | PSC Independent Member (Qualitative) | Research Fellow in the Centre for Academic Primary Care, University of Bristol |
| **Graeme MacLennan** | PSC Independent Member (Statistics) | Senior Research Fellow in Statistics, University of Aberdeen |
| **Richard Grieve** | PSC Independent Member  (Health Economics) | Professor of Health Economics Methodology, London School of Hygiene and Tropical Medicine |
| **Frank Sullivan** | PSC Independent Member  (Primary Care) | Professor of Primary Care Medicine, University of St Andrews |
| **Abigail Dennington-Price** | PSC Independent Member  (Patient Contributor) | CRS patient and member of the public |
| **John Abrey** | PSC Independent Member  (Patient Contributor) | CRS patient and member of the public |
| **Claire Hopkins** | MACRO Co-Chief Investigator | ENT Consultant Guy’s and St. Thomas’ NHS Foundation Trust, Professor Kings College London |
| **Carl Philpott** | MACRO Co-Chief Investigator | ENT Consultant James Paget University Hospital,  Professor University of East Anglia |
| **Helen Blackshaw** | MACRO Programme Manager | Senior Research Associate evidENT, UCL |
| **Steffi le Conte** | MACRO Trial Manager | SITU, University of Oxford |
| **Jonathan Cook** | MACRO Lead Trial Statistician | SITU, University of Oxford |

| **Data Safety and Monitoring Committee** | | |
| --- | --- | --- |
| **Alan Montgomery** | DSMC Independent Chair | Professor of Medical Statistics and Clinical Trials, Faculty of Medicine & Health Sciences, University of Nottingham |
| **Lee Middleton** | DSMC Independent Member | Senior Medical Statistician, University of Birmingham |
| **Hisham Mehanna** | DSMC Independent Member | Professor of Head and Neck Surgery, University of Birmingham |

# Appendix 2


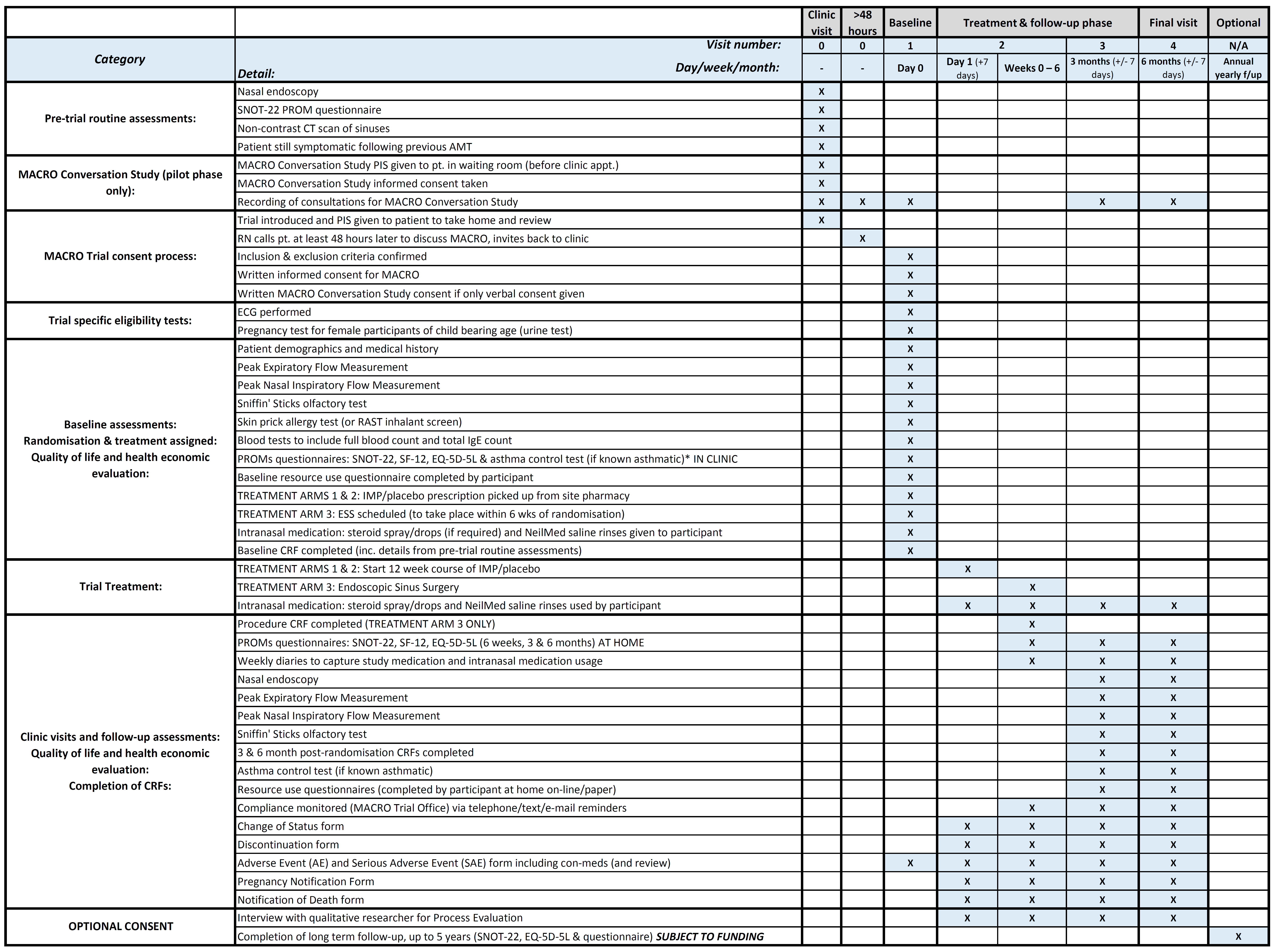

Supplement: Supplementary file 1 — Appendix 1. Trial registration, protocol version, funding details, roles and responsibilities. Appendix 2. Schedule of enrolment, interventions, and assessments (Spirit guidelines figure). (DOCX 1089 kb) [file 13063_2019_3314_MOESM1_ESM.docx]
